# Unveiling hidden multipolar orders with magnetostriction

**DOI:** 10.1038/s41467-019-11913-3

**Published:** 2019-09-09

**Authors:** Adarsh S. Patri, Akito Sakai, SungBin Lee, Arun Paramekanti, Satoru Nakatsuji, Yong Baek Kim

**Affiliations:** 1 Department of Physics and Centre for Quantum Materials, University of Toronto, Toronto, ON M5S 1A7 Canada; 2 Institute for Solid State Physics, University of Tokyo, Kashiwa, Chiba 277-8581 Japan; 3 CREST, Japan Science and Technology Agency (JST), 4-1-8 Honcho Kawaguchi, Saitama, 332-0012 Japan; 4 Department of Physics, Korea Advanced Institute of Science and Technology, Daejeon, 34141 Korea

**Keywords:** Magnetic properties and materials, Phase transitions and critical phenomena

## Abstract

Broken symmetries in solids involving higher order multipolar degrees of freedom are historically referred to as “hidden orders” due to the formidable task of detecting them with conventional probes. In this work, we theoretically propose that magnetostriction provides a powerful and novel tool to directly detect higher-order multipolar symmetry breaking—such as the elusive octupolar order—by examining scaling behaviour of length change with respect to an applied magnetic field *h*. Employing a symmetry-based Landau theory, we focus on the family of Pr-based cage compounds with strongly correlated *f*-electrons, Pr(Ti,V,Ir)_2_(Al,Zn)_20_, whose low energy degrees of freedom are purely higher-order multipoles: quadrupoles ${\cal{O}}_{20,22}$ and octupole ${\cal{T}}_{xyz}$. We demonstrate that a magnetic field along the [111] direction induces a distinct linear-in-*h* length change below the octupolar ordering temperature. The resulting “magnetostriction coefficient” is directly proportional to the octupolar order parameter, thus providing clear access to such subtle order parameters.

## Abstract (web summary)

Higher-order multipolar phases are unusual states that can form in correlated materials and are difficult to observe as they do not directly couple to conventional probes. Patri et al. theoretically show that angle-dependent magnetostriction measurements can probe quadrupolar and octupolar ordering.

## Introduction

In crystalline solids, the combination of spin–orbit coupling and crystal electric fields places strong constraints on the shape of localized electronic wavefunctions^1^. The quantum mechanically defined multipole moments provide a useful measure of the resulting complex angular distribution of the magnetization and charge densities^2,3^. Most conventional broken symmetry phases in solids involve the magnetic dipole moment of the electron. Remarkably, a large class of strongly correlated electron materials display non-trivial higher order multipolar moments, e.g., quadrupolar or octupolar moments, whose fluctuations and ordering leads to a rich variety of phases, such as quadrupolar heavy Fermi liquids^4–6^, superconductivity^7–9^, and unusual multipolar symmetry-broken phases^2,3,10–13^. While multipolar ordered phases fall under the purview of the celebrated Landau paradigm of symmetry-broken phases, they have been termed as so-called ‘hidden orders’: mysterious phases of matter whose orderings are invisible to conventional local probes (such as neutron scattering or magnetic resonance), but are remarkably still known to exist as their onset triggers non-analytic signatures in thermodynamic measurements^4,14–17^. Studying the mysterious ordering patterns of higher order multipoles is also often rendered challenging since they typically coexist with conventional dipolar moments. Examples of such symmetry breaking which are of great interest include spin-nematic order^18^ in spin *S* ≥ 1 quantum magnets, quadrupolar charge order in transition metal oxides, and higher multipolar order in actinide dioxides, such as NpO_2_^19^, and *f*-electron heavy fermion materials^20^, such as URu_2_Si_2_^21–29^ and UBe_13_^30–32^. The quest to probe such orders has led to novel experimental techniques, e.g., elasto-resistivity^33–35^ to elucidate the quadrupolar order associated with orbital nematicity in the iron pnictides. A broad understanding of the nature of these symmetry-broken phases, and means to definitively demonstrate their existence, has proven to be a challenging, yet stimulating, endeavour for both theory and experiment.

Our work is motivated by a recent series of experiments on the Pr-based cage compounds Pr(Ti,V,Ir)_2_(Al,Zn)_20_ which form an ideal setting to study multipolar moments and associated hidden orders^8,17,36–38^. In these systems, the 4*f*^2^ electrons of Pr^3+^ ions subject to CEFs host a ground non-Kramers doublet with solely higher-order moments: quadrupoles (${\cal{O}}_{20}$ and ${\cal{O}}_{22}$) and octupole (${\cal{T}}_{xyz}$)^14,39^. Uncovering and understanding the pattern of multipolar ordering across this family of materials remains an important open problem.

The nature of the quadrupolar ordering in these cage compounds has been indirectly examined with a few techniques^40,41^ such as ultrasound experiments^42–45^ (indicating softening of elastic modulus at quadrupolar ordering temperature, $T_{\cal{Q}}$), as well as NMR measurements (where the magnetic field-induced dipole moment is strongly dependent on the underlying quadrupolar phase^46^). More recently, magnetostriction and thermal expansion strain experiments^47^ have also lent themselves as possible probes to study the transitions and the underlying quadrupolar phase. By contrast, the octupolar ordered state has continued to remain an elusive phase of matter, with only indirect hints of its existence from NMR^48^ and *μ*SR^49^ measurements, but as yet no direct probe to reveal its existence^50^. More recently, some of us (A.S. and S.N., unpublished) have begun experiments to study angle-dependent magnetostriction, the change in sample length induced by a magnetic field which can point along various crystalline directions, in a wide class of materials with multipolar degrees of freedom.

In this work, motivated by these experiments, we theoretically discuss how magnetostriction provides a novel means to directly probe multipolar order parameters. The central observation of this paper is that an applied magnetic field allows for a linear coupling between lattice strain fields and a uniform octupole moment which depends strongly on the applied field direction. In the absence of a dipolar moment, this enables measurements of the magnetostriction to directly reveal the hidden octupolar order parameter. We investigate such field-scaling behaviour of the magnetostriction for various magnetic field directions by employing a symmetry-based Landau theory, which allows us to highlight the universal aspects of the physics and to reveal its applicability to a broader class of materials. Specifically, our Landau theory permits both antiferro-quadrupolar ordering (AF${\cal{Q}}$) and ferro-octupolar ordering (F${\cal{O}}$), and we examine our theory along different field directions in three temperature regimes. Denoting the quadrupolar and octupolar transition temperatures as $T_{\cal{Q}}$ and $T_{\cal{O}}$, respectively, we consider the regimes (i) the paramagnetic phase above both transition temperatures ($T \,> \, T_{\cal{Q}},T_{\cal{O}}$), (ii) intermediate temperatures ($T_{\cal{O}} \,< \,T \,< \,T_{\cal{Q}}$) where the system exhibits pure quadrupolar order, and (iii) below both ordering temperatures ($T \,< \,T_{\cal{Q}},T_{\cal{O}}$) where the system features coexisting quadrupolar and octupolar orders. We make definite predictions for all possible combinations of length change and magnetic field directions, which can be tested in future experiments.

## Results

### Magnetostriction as a probe of multipolar ordering

Our studies predict a linear-in-*h* scaling behaviour for particular length changes, for $T \,<\, T_{\cal{O}}$. The coefficient of the linear-in-*h* term, i.e. the “magnetostriction coefficient”, is directly proportional to the ordered ferro-octupolar moment, thus providing a clear and distinct means to directly probe this order parameter. This linear-in-*h* behaviour appears for magnetic fields applied along the [111] and [100] directions. For other magnetic field (and length change) directions, we predict the signature of quadrupolar ordering as a constant plus quadratic-in-*h* scaling behaviour in the length change; although the scaling behaviour explicitly involves the F${\cal{Q}}$ order, the AF${\cal{Q}}$ order parameter can be inferred from the F${\cal{Q}}$, as it scales (to leading order) as the square root of the F${\cal{Q}}$ order parameter. We present our theoretical predictions for the scaling behaviours in Table 1 for a variety of magnetic field and length change directions. A quick way to see this linear-in-*h* result is to note that the elastic energy of a strained cubic crystal is given by^51,52^

1 $$F_{{\mathrm{lattice}}} =	 \frac{{c_{\mathrm{B}}}}{2}\left( {\epsilon _{\mathrm{B}}^2} \right) + \frac{{c_{11} \,-\, c_{12}}}{2}\left( {\epsilon _{\mu} ^2 + \epsilon _{\nu} ^2} \right)\\ 	+ \frac{{c_{44}}}{2}\left( {\epsilon _{xy}^2 + \epsilon _{yz}^2 + \epsilon _{xz}^2} \right)\;,$$

where the crystal’s deformation is described by the components of the strain tensor $\epsilon _{ik}$, and *c*_*ij*_ is the elastic modulus tensor describing the stiffness of the crystal. Note that we use the normal modes of the cubic lattice to write the elastic energy in this elegant form. Here *c*_B_ is the bulk modulus, $\epsilon _{\mathrm{B}} \equiv \epsilon _{xx} + \epsilon _{yy} + \epsilon _{zz}$ is the volume expansion of the crystal, $\epsilon _\nu \equiv (2\epsilon _{zz} - \epsilon _{xx} - \epsilon _{yy})/\sqrt 3$ and $\epsilon _\mu \equiv (\epsilon _{xx} - \epsilon _{yy})$ are lattice strains that transform as the Γ_3*g*_ irreducible representation (irrep.) of the *O*_*h*_ group, and the off-diagonal strain components transform as the Γ_5*g*_ irrep. of *O*_*h*_ group. The subscript *g* indicates even under time-reversal and spatial inversion (parity). Knowing $\epsilon _{ij}$ determines the fractional length change along the $\hat {\bf{\ell }}$-axis via $(\Delta L/L)_{ {\bf{\ell }}} = \mathop {\sum}\limits_{ij} {\epsilon _{ij}} \hat \ell _i\hat \ell _j$, where $\hat \ell _i$ is the *i*^th^ component of unit vector $\hat {\bf{\ell }}$; Supplementary Note 1 provides a more detailed discussion on this expression. As discussed below, an applied magnetic field enables a linear coupling between the strain field and the time-reversal-odd ferro-octupolar moment, *m*, via a term in the free energy $\Delta F = - g_{\cal{O}}m(\epsilon _{yz}h_x + \epsilon _{xz}h_y + \epsilon _{xy}h_z)$, with a couplin*g* constant $g_{\cal{O}}$. Minimizing *F*_lattice_ + Δ*F* with respect to the strain, we find $\epsilon _{xy} \propto (g_{\cal{O}}/c_{44})mh_z$, and cyclically for $\epsilon _{yz},\epsilon _{xz}$, while diagonal components of the strain tensor vanish. As a representative example, take a [111] field, where $h_i = h/\sqrt 3$, this leads to $(\Delta L/L)_{(1,1,1)} = (\epsilon _{xy} + \epsilon _{yz} + \epsilon _{xz})/3$ and so (Δ*L*/*L*)_(1,1,1)_ ∝ ($g_{\cal{O}}$/*c*_44_)*mh*. This direct relation between the linear-in-*h* magnetostriction coefficient and the ferro-octupolar order parameter for a magnetic field along the [111] direction is one of the central results of our paper. Furthermore, we predict a characteristic hysteresis in the octupolar moment and the associated parallel length change as a function of magnetic field, arising from the symmetry-allowed cubic-in-*h* coupling of the magnetic field to the octupolar moment. Very recent (unpublished) experiments on PrV_2_Al_20_ indeed appear to find a hysteretic linear-in-field magnetostriction, for a [111] magnetic field, below a transition at T* ≈ 0.65 K. Our theoretical results for magnetostriction in the presence of octupolar order thus lend strong support to the idea that this approach, pursued in recent experiments performed by some of us (A.S. and S.N., unpublished), herald the unambiguous discovery of octupolar order.

**Table 1:** Scaling relation for relative length change of system ${\mathrm{\Delta }}L/L_{\mathbf{\ell }}$ along direction ${\mathbf{\ell }}$ for magnetic field applied along $\widehat {\mathbf{n}}$ direction

Magnetic field, h = *h* $\widehat {\mathbf{n}}$	ℓ	${\mathrm{\Delta }}L/L_{{\ell }}$ scaling: $T > T_{\cal{Q}},T_{\cal{O}}$	${\mathrm{\Delta }}L/L_{{\ell }}$ scaling: $T_{\cal{O}} < T < T_{\cal{Q}}$	${\mathrm{\Delta }}L/L_{{\ell }}$ scaling: $T < T_{\cal{Q}},T_{\cal{O}}$
$\widehat {\mathbf{n}}$ = [100]	${\mathbf{\ell }} = (1,0,0)$	*κ*_1_ *h* ^2^	Φ_1_ + *κ*_1_*h*^2^	Φ_1_ + *κ*_1_*h*^2^
	${\mathbf{\ell }} = (0,1, \pm 1)$	*κ*_1_ *h* ^2^	Φ_1_ + *κ*_1_*h*^2^	${\mathrm{\Phi }}_1 \pm {\cal{M}}h + \kappa _1h^2$
$\widehat {\mathbf{n}} = \frac{1}{{\sqrt 2 }}[110]$	${\mathbf{\ell }} = (1,1,0)$	$\frac{1}{2}\gamma h^2 + \frac{1}{2}\kappa _2h^2$	${\mathrm{\Phi }}_2 + \frac{1}{2}\gamma h^2 + \frac{1}{2}\kappa _2h^2$	${\mathrm{\Phi }}_2 + \frac{1}{2}\gamma h^2 + \frac{1}{2}\kappa _2h^2$
	${\mathbf{\ell }} = (1, - 1,1)$	$- \frac{1}{3}\gamma h^2$	$- \frac{1}{3}\gamma h^2$	$- \frac{1}{3}\gamma h^2$
	${\mathbf{\ell }} = ( - 1,1,2)$	$- \frac{1}{6}\gamma h^2 + \frac{1}{2}\kappa _2h^2$	${\mathrm{\Phi }}_2 - \frac{1}{6}\gamma h^2 + \frac{1}{2}\kappa _2h^2$	${\mathrm{\Phi }}_2 - \frac{1}{6}\gamma h^2 + \frac{1}{2}\kappa _2h^2$
$\widehat {\mathbf{n}} = \frac{1}{{\sqrt 3 }}[111]$	${\mathbf{\ell }} = (1,1,1)$	$\frac{2}{3}\gamma h^2$	$\frac{2}{3}\gamma h^2$	$\frac{2}{{\sqrt 3 }}{\cal{M}}h + \frac{2}{3}\gamma h^2$
	${\mathbf{\ell }} = (1, - 1,0)$	$- \frac{1}{3}\gamma h^2 + \frac{1}{3}{\kappa} _2h^2$	${\mathrm{\Phi }}_2 - \frac{1}{3}\gamma h^2 + \frac{1}{3}{\kappa} _2h^2$	${\mathrm{\Phi }}_2 - \frac{1}{{\sqrt 3 }}{\cal{M}}h - \frac{1}{3}\gamma h^2 + \frac{1}{3}{\kappa} _2h^2$
	${\mathbf{\ell }} = (1,1, - 2)$	$- \frac{1}{3}\gamma h^2 + \frac{1}{3}{\kappa} _2h^2$	${\mathrm{\Phi }}_2 - \frac{1}{3}\gamma h^2 + \frac{1}{3}{\kappa} _2h^2$	${\mathrm{\Phi }}_2 - \frac{1}{{\sqrt 3 }}{\cal{M}}h - \frac{1}{3}\gamma h^2 + \frac{1}{3}{\kappa} _2h^2$

For each $\widehat {\mathbf{n}}$, we present the length change parallel and (the two) perpendicular directions with respect to $\widehat {\mathbf{n}}$. The F${\cal{Q}}$ moment term is expressed as $\overline {g_{\cal{Q}}} |\phi | {\equiv} \left( {{\Phi} _{1,2} + {\kappa} _{1,2}h^2} \right)$ due to the even-in-*h* behaviour of the quadrupolar moment, where Φ_1,2_ is the zero magnetic field quadrupolar moment which arises due to AF${\cal{Q}}$ spontaneously ordering. Here, the two types of $\overline {g_{\cal{Q}}}$ (and *κ*_1,2_, Φ_1,2_) include the complex angle-dependent parts (*α*) and the quadrupolar–lattice strain coupling; as described in Supplementary Note 6, there are two possible combinations of the complex angle dependency in $\overline {g_{\cal{Q}}}$, which we denote by the subscripts 1, 2. Since Φ_1,2_,*κ*_1,2_ arise from the parasitic F${\cal{Q}}$ moment, they are diminutive, as compared with the conduction electrons’ term (*γ* ≡ *γ*_c_/*c*_44_). ${\cal{M}} \equiv \frac{{g_{\cal{O}}}}{{c_{44}}}m$ is a re-defined octupolar moment, including the octupolar–lattice strain coupling.

The theoretical roadmap which gives rise to this striking result requires (i) the Landau free energy of the multipolar moments, and (ii) coupling between the multipolar moments and lattice strain. We present these key ingredients below.

### Landau theory of multipolar order

We present in this section, for the sake of self-containedness and to specify our notation, the Landau theory of multipolar order first introduced in ref. ^51^. We focus on key aspects of the model here, and relegate the symmetry-based derivation as well as the complete form of the free energy to Methods; the symmetry transformations of the multipolar moments are given in Supplementary Note 2.

The 4*f*^2^ electrons of Pr^3+^ ions in the family of rare-earth metallic compounds Pr(Ti,V,Ir)_2_(Al,Zn)_20_ reside on a diamond lattice of cubic space group Fd$\bar 3$m. Surrounding each Pr^3+^ ion is a Frank-Kasper (FK) cage (16 Al atom polyhedra). The crystalline electric field (CEF) of this FK cage, with *T*_*d*_ point group symmetry, splits the *J* = 4 multiplet of the 4*f*^2^ electrons. The ground states are experimentally found to be a non-Kramers doublet, and they transform as the basis states of the Γ_3*g*_ irrep. of *T*_*d*_; here the subscript g(erade) and u(ngerade) denote even and odd under time-reversal, respectively. Moreover, this doublet is energetically well separated from the excited states, and so for energies much lower than this gap (≳50 K^4^), the Γ_3*g*_ doublets form an ideal basis to describe the low energy degrees of freedom. The Γ_3*g*_ doublets can give rise to time-reversal even quadrupolar moments ${\cal{O}}_{22} = \frac{{\sqrt 3 }}{2}(J_x^2 - J_y^2)$ and ${\cal{O}}_{20} = \frac{1}{2}(2J_z^2 - J_x^2 - J_y^2)$ which transform as Γ_3*g*_, as well as a time-reversal odd octupolar moment ${\cal{T}}_{xyz} = \frac{{\sqrt {15} }}{6}\overline {J_xJ_yJ_z}$ which transforms as Γ_2*u*_ (where the overline represents the fully symmetrized product). Using the constructed pseudospin basis ({|↑〉, |↓〉}) from the Γ_3*g*_ doublets, allows the multipolar moments to be neatly denoted by an effective pseudospin-1/2 operator ***τ*** = (*τ*^*x*^, *τ*^*y*^, *τ*^*z*^)

2 $$\tau ^x = - \frac{1}{4}{\cal{O}}_{22},\quad \tau ^y = - \frac{1}{4}{\cal{O}}_{20},\quad \tau ^z = \frac{1}{{3\sqrt 5 }}{\cal{T}}_{xyz}.$$

The perpendicular component of the pseudospin vector ***τ***^⊥^ ≡ (*τ*^*x*^, *τ*^*y*^) denotes the quadrupole moments, while *τ*^*z*^ denotes the octupolar moment. We also define the raising/lowering pseudospin operators *τ*^±^ = *τ*^*x*^ ± i*τ*^*y*^.

The ordering of these multipolar degrees of freedom acts as a mean field on the pseudospins, and breaks the degeneracy of the non-Kramers doublet. In order to describe these pseudospin-symmetry-broken phases, we resort to a Landau theory approach, focussing on the following order parameters,

3 $$\begin{array}{l}\phi \equiv \langle \tau _{\mathrm{A}}^ + \rangle + \langle \tau _{\mathrm{B}}^ + \rangle ,\\ \tilde \phi \equiv \langle \tau _{\mathrm{A}}^ + \rangle - \langle \tau _{\mathrm{B}}^ + \rangle ,\\ m \equiv \langle \tau _{\mathrm{A}}^z\rangle + \langle \tau _{\mathrm{B}}^z\rangle ,\\ \tilde m \equiv \langle \tau _{\mathrm{A}}^z\rangle - \langle \tau _{\mathrm{B}}^z\rangle ,\end{array}$$

Here, angular brackets 〈...〉 denote thermal averages, while the A, B subscripts denote the two sublattices of the diamond lattice. The complex scalars *ϕ* and $\tilde \phi$ describe ferroquadrupolar (F${\cal{Q}}$) and anti-ferroquadrupolar (AF${\cal{Q}}$) orders, respectively, while the real scalars *m* and $\tilde m$ denote the ferro-octupolar (F${\cal{O}}$) and anti-ferrooctupolar (AF${\cal{O}}$) order parameters. We henceforth use the convention of $\tilde \phi = |\tilde \phi |{\mathrm{e}}^{{\mathrm{i}}\tilde \alpha }$ and *ϕ* = |*ϕ*|e^i*α*^ for the complex order parameters.

**Fig. 1:**
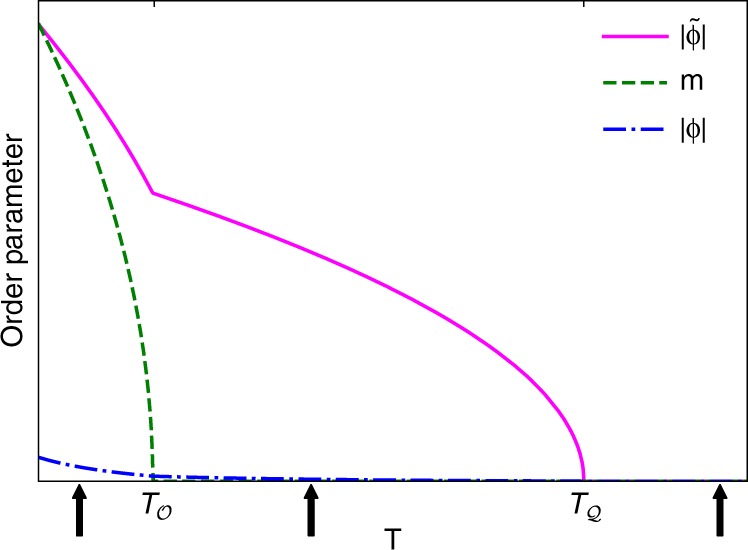
Phase diagram at zero magnetic field [*h* = 0]. The temperature regimes studied in Table 1 and in Supplementary Note 4 are denoted by vertical arrows at: $T \,<\, T_{\cal{Q}},T_{\cal{O}}$, $T_{\cal{O}} \,< \,T \,<\, T_{\cal{Q}}$, and $T \,> \, T_{\cal{Q}},T_{\cal{O}}$. The order parameters for AF${\cal{Q}}$, F${\cal{O}}$, and F${\cal{Q}}$ are denoted by $|\tilde \phi |$, *m*, and |*ϕ*|, respectively. AF${\cal{Q}}$ and F${\cal{O}}$ spontaeously order at $T_{\cal{Q}}$ and $T_{\cal{O}}$, respectively

In this work, we focus on a system where the primary order parameters are AF${\cal{Q}}$ and F${\cal{O}}$. As discussed in previous works^53,54^, the Landau theory of a system with AF${\cal{Q}}$ order necessarily admits a ‘parasitic’ secondary order parameter F${\cal{Q}}$. Such mixing does not occur for the octupolar order parameter; motivated by explaining the experiments performed by some of us (A.S. and S.N., unpublished) on PrV_2_Al_20_, we choose to work with only F${\cal{O}}$ order and ignore the AF${\cal{O}}$ order parameter. We can thus construct our Landau theory using the order parameters *ϕ*, $\tilde \phi$, and *m*, based on the local *T*_*d*_ symmetry instilled by the FK cage, $F_{{\cal{Q}},{\cal{O}}}[\phi ,\tilde \phi ,m] = F_{\tilde \phi } + F_m + F_\phi + F_{\tilde \phi ,\phi ,m}$. Here, the free energies $F_{\tilde \phi }$, *F*_*m*_, and *F*_*ϕ*_ denote the independent free energies of the AF${\cal{Q}}$, F${\cal{O}}$, and F${\cal{Q}}$ orders, and $F_{\tilde \phi ,\phi ,m}$ describes the interactions between the different multipolar order parameters. Figure 1 shows the zero magnetic field phase diagram, depicting both quadrupolar and octupolar transitions; with two primary order parameters AF${\cal{Q}}$ (and its accompanying parasitic F${\cal{Q}}$ moment) and F${\cal{O}}$ ordering at critical temperatures of $T_{\cal{Q}}$ and $T_{\cal{O}}$, respectively. The ‘kink’ in the AF${\cal{Q}}$ (as well as F${\cal{Q}}$) at the octupolar ordering temperature reflects the non-analytic behaviour of the octupolar moment at its critical temperature. We present in Supplementary Note 3 the values of the Landau parameters used for Fig. 1.

In order to study magnetostriction, it is important to understand how the magnetic field couples to the multipole moments. Due to the lack of magnetic dipole moment supported by the Γ_3*g*_ doublet, the magnetic field does not couple linearly to the states. One can derive the low energy magnetic field Hamiltonian by performing second-order perturbation theory in **h** **·** **J**, where the low energy subspace is spanned by the Γ_3*g*_ doublet, and the high energy subspace is spanned by the excited triplets Γ_4,5_. This leads to $F_{{\mathrm{mag}}}[\phi ,\tilde \phi ]$, which involves the quadrupolar moments coupling quadratically to the magnetic field, ~*h*^2^*τ*^*x*,*y*^. The coupling of the magnetic field to the octupole moment (after performing third-order perturbation theory) is of the form ~*h*_*x*_*h*_*y*_*h*_*z*_*τ*^*z*^. The ${\cal{O}}(h^3)$ term  is neglected at this stage, and its role is revived in the discussion of hysteresis.

### Symmetry allowed coupling of multipoles to lattice modes

We now turn our attention to the problem of coupling the lattice normal modes of the cubic crystal to the multipolar moments. We recall that the cubic crystal structure supports macroscopic normal modes that transform as irreps. of *O*_*h*_, while the Landau free energy of the multipolar moments (*F*) is constructed subject to symmetries of the local *T*_*d*_ environment. The symmetry constraints on *F* ensure that in principle only select normal modes of the crystal that transform as the irreps. of *T*_*d*_ are permitted to couple to the multipolar moments. In the present case, all the cubic normal modes presented in Eq. (1) also transform as irreps. under *T*_*d*_ (as can be explicitly verified), and so all of the aforementioned strain modes can participate in the coupling.

### Coupling of quadrupolar moment to lattice strain

Coupling between the quadrupolar moments and the lattice normal modes appears as a natural choice, as the quadrupolar moments and the lattice strains are both even under time-reversal. Moreover, both the normal modes $\{ \epsilon _\mu ,\epsilon _\nu \}$ and the quadrupolar moments $\{ {\cal{O}}_{22},{\cal{O}}_{20}\}$ transform as Γ_3*g*_ irreps. of *T*_*d*_ (the aforementioned lattice normal modes also transform as Γ_3*g*_ in *O*_*h*_, as *T*_*d*_ is a subgroup of *O*_*h*_). This similarity in how they transform under *T*_*d*_ allows a linear coupling between the aforesaid lattice normal modes and quadrupolar moments. Thus, the Landau free energy of the multipolar moments gets augmented by the following lattice elastic energy and coupling terms to quadrupolar moments,

4 $$ F_{{\mathrm{strain}},{\cal{Q}}} =	 \frac{{c_{11} - c_{12}}}{2}\left( {\epsilon _\mu ^2 + \epsilon _\nu ^2} \right) - g_{\cal{Q}}\epsilon _\mu \left[ {\langle \tau _{\mathrm{A}}^x\rangle + \langle \tau _{\mathrm{B}}^x\rangle } \right]\\ 	-\, g_{\cal{Q}}\epsilon _\nu \left[ {\langle \tau _{\mathrm{A}}^y\rangle + \langle \tau _{\mathrm{B}}^y\rangle } \right],$$

where $g_{\cal{Q}}$ is the coefficient of coupling between the quadrupolar moments and lattice strain tensors. Note that we include the coupling of the lattice strain to the quadrupole moment on each sublattice. Using the definition of the order parameter *ϕ* from Eq. (3), and minimizing $F_{{\mathrm{strain}},{\cal{Q}}}$ with respect to $\epsilon _\mu ,\epsilon _\nu$ yields the total strain for each normal mode

5 $$\begin{array}{l}\epsilon _\mu = \frac{{g_{\cal{Q}}}}{{(c_{11} \,-\, c_{12})}}|\phi |\cos \alpha \;,\\ \epsilon _\nu = \frac{{g_{\cal{Q}}}}{{(c_{11} \,-\, c_{12})}}|\phi |\sin \alpha \;.\end{array}$$

Substituting these expressions back into Eq. (4), we find that the strain-optimized $F_{{\mathrm{strain}},{\cal{Q}}}[\phi ] = - \frac{{g_{\cal{Q}}^2}}{{2(c_{11} - c_{12})}}|\phi |^2$ renormalizes the mass term of the F${\cal{Q}}$ order.

### Coupling of octupolar moment to lattice strain

A direct linear coupling between the octupolar moment ${\cal{T}}_{xyz}$ and the lattice normal modes is not permitted, as the octupolar moment is odd under time-reversal. However, this potential difficulty can be alleviated by the introduction of the time-reversal odd magnetic field *h* which assists in the coupling between the lattice degrees of freedom and octupolar moment. Thus, the Landau free energy of the multipolar moments gets augmented by the following lattice elastic energy and the coupling terms to the octupolar moments,

6 $$F_{{\mathrm{strain}},{\cal{O}}} =	\frac{{c_{44}}}{2}\left( {\epsilon _{xy}^2 + \epsilon _{yz}^2 + \epsilon _{xz}^2} \right)\\ 	-\, g_{\cal{O}}m\left[ {h_x\epsilon _{yz} + h_y\epsilon _{xz} + h_z\epsilon _{xy}} \right]\\ 	-\, {\gamma} _{\mathrm{c}}\left[ {h_xh_y\epsilon _{xy} + h_xh_z\epsilon _{xz} + h_yh_z\epsilon _{yz}} \right],$$

where we use the definition of *m* from Eq. (3), and $g_{\cal{O}}$ is the coefficient of coupling between the octupolar moment and lattice strain. We also include another symmetry-allowed direct coupling between the magnetic field and the same lattice normal modes (with proportionality constant *γ*_c_, equivalent on both sublattices). Physically, this kind of term could arise from the independent coupling of the magnetic field and lattice strain to the conduction electrons (and after integrating out the conduction electrons). We discuss in Supplementary Note 5 how the numerical values of these coupling constants can be obtained from experimental observations in conjunction with our theoretical predictions.

Minimizing with respect to the lattice degrees of freedom yields the following expressions for the (total) lattice strains

7 $$\begin{array}{l}\epsilon _{xy} = \left( {\frac{{g_{\cal{O}}h_z}}{{c_{44}}}} \right)m + {\gamma} _{\mathrm{c}}\frac{{h_xh_y}}{{c_{44}}}\;,\\ \epsilon _{xz} = \left( {\frac{{g_{\cal{O}}h_y}}{{c_{44}}}} \right)m + {\gamma} _{\mathrm{c}}\frac{{h_xh_z}}{{c_{44}}}\;,\\ \epsilon _{yz} = \left( {\frac{{g_{\cal{O}}h_x}}{{c_{44}}}} \right)m + {\gamma} _{\mathrm{c}}\frac{{h_yh_z}}{{c_{44}}}\;.\end{array}$$

Substituting the expression for the minimized lattice strains from Eq. (7) into Eq. (6) yields $F_{{\mathrm{strain}},{\cal{O}}}[m]$, where the mass term of the octupolar moment is renormalized by a term quadratic in *h*; it also introduces an ${\cal{O}}(h^3)$ coupling term between the octupolar moment and the magnetic field, which renormalizes the coefficient of the already present *h*_*x*_*h*_*y*_*h*_*z*_*m* from third-order in perturbation theory in **h** ⋅ **J**.

### Length change under magnetic field along various directions

Equipped with the necessary ingredients in the previous subsections, we can now examine the relative length change, Δ*L*/*L*, for magnetic fields applied along [100], [110], [111] directions and examine the scaling in magnetic field strength, *h*. For the sake of clarity, we stress that we consider here the complete Landau theory of multipolar moments coupled to lattice strain fields (after having integrated out the lattice degrees of freedom): $F[\phi ,\tilde \phi ,m] = F_{{\cal{Q}},{\cal{O}}}[\phi ,\tilde \phi ,m] + F_{{\mathrm{mag}}}[\phi ,\tilde \phi ] + F_{{\mathrm{strain}},{\cal{Q}}}[\phi ] + F_{{\mathrm{strain}}, {\cal{O}}}[m]$. The scaling relations can be inferred by substituting the expressions for the (extremized) strain in Eqs. (5) and (7) into $(\Delta L/L)_{ {\bf{\ell }}} = \mathop {\sum}\limits_{ij} {\epsilon _{ij}} \hat \ell _i\hat \ell _j$. We stress that from Eq. (7), the off-diagonal strain components involve the octupolar moment; thus to have any possibility of observing *m*, it requires length change expressions that are not along purely the crystal axes [100], [010], [001]. We summarize the key results in Table 1. Taking the example of length changes along the (1, ±1, 1) direction we have

8 $$\left( {\frac{{\Delta L}}{L}} \right)_{(1, \pm 1,1)}=	 \frac{{\epsilon _{\mathrm{B}}}}{3} + \frac{{2\left( { \pm \epsilon _{xy} \,\pm\, \epsilon _{yz} \,+\, \epsilon _{xz}} \right)}}{3}\\ =	 \frac{1}{3}\epsilon _{\mathrm{B}} + \frac{{2g_{\cal{O}}m}}{{3c_{44}}}\left[ { \pm h_z \pm h_x + h_y} \right]\\ 	+ \frac{{2{\gamma} _{\mathrm{c}}}}{{3c_{44}}}\left[ { \pm h_xh_y \pm h_yh_z + h_xh_z} \right].$$

This equation has a striking conclusion as it pertains to observing hidden order. The mysterious octupolar moment can now be determined (up to a proportionality constant) by measuring the slope of the linear-in-*h* behaviour of the length change both parallel and perpendicular to magnetic fields applied along the [111] direction. This provides a clear signature for the onset of the octupolar ordering as well as a means to study the general behaviour of the octupolar moment (up to a proportionality constant) with respect to other external variables such as temperature, *T*. Moreover, we discover that length change parallel to the magnetic field along [111] has (negative) twice the slope of the linear-in-*h* term and (negative) twice the quadratic background as the length changes perpendicular ${\bf{\ell }} = (1, - 1,0),(1,1, - 2)$ to the field [111]. Furthermore, from Table 1, the octupolar moment analogously appears in the length change perpendicular to the magnetic field along the [100] direction. Indeed, the sign of linear-in-*h* coefficient flips for the two presented orthogonal directions. All of these provide distinct means to validate the theory.

**Fig. 2:**
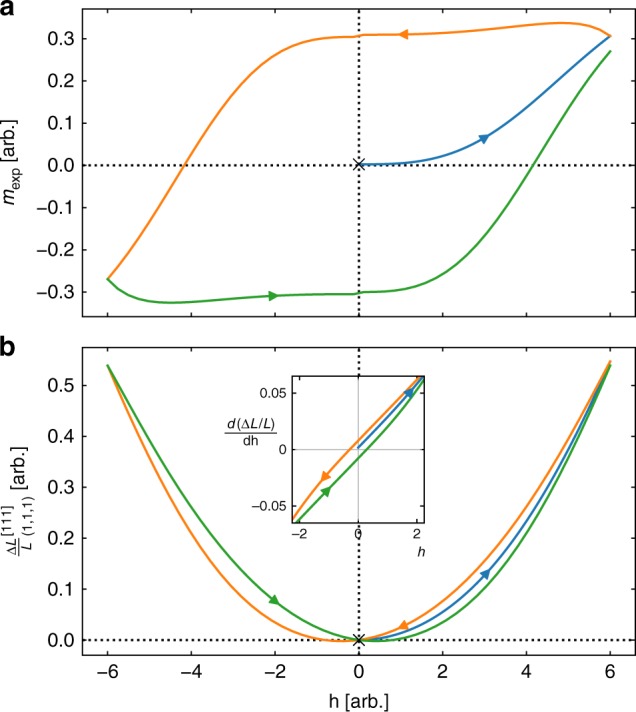
Hysteresis for **h** || [111]. **a** Total octupolar order parameter (*m*_exp_) versus magnetic field strength (*h*) along [111] direction demonstrating hysteresis for $T \,<\, T_{\cal{Q}},T_{\cal{O}}$. The initial condition is denoted by ‘×’ in Fig. 2a, b. **b** Length change along (1, 1, 1) direction demonstrating hysteresis, using the solution of (**a**), and taking *γ*_c_ = 0.8. Inset: Derivative of length change along (1, 1, 1) direction with respect to magnetic field strength ($\frac{{d(\Delta L/L)}}{{dh}}$) versus magnetic field. The linear-in-*h* scaling of Δ*L*/*L* is reflected as a constant *y*-intercept here

Next, for magnetic fields along the [110] direction, we find that the length changes parallel, ${\bf{\ell }} = (1,1,0)$, and perpendicular, ${\bf{\ell }} = (1, - 1,1),( - 1,1,2)$, to the field are purely quadratic-in-*h* and do not possess a linear-in-*h* scaling behaviour. Thus, these length changes (for this choice of magnetic field) do not provide information about the octupolar moment; the quadratic in *h* behaviour arises from the conduction electrons and/or the quadrupolar moment. We provide in Supplementary Note 4 a justification of the scaling behaviours of the multipolar moments, and in Supplementary Note 6 the corresponding general length change expressions. We note that the scaling behaviours presented here and in Supplementary Note 6 neglect the cubic-in-*h* coupling, which breaks the ℤ_2_ symmetry (*m* ↔ −*m*) of the octupolar moment. This introduces a ‘flip’ in the octupolar moment at *h* = 0 (and at $T < T_{\cal{O}}$ where *m* has spontaneously ordered, i.e. *m* ≠ 0): for *h* = 0^+^, the +|*m*| solution is ‘chosen’, and as we crossover to *h* = 0^−^, the now physically distinct −|*m*| solution is ‘chosen’ (this is seen in Fig. 2). A similar phenomena is observed in usual ferromagnetism, below the ordering temperature. The neglect of this term is due to the consideration of weak, perturbative magnetic fields in this study. It is likely that this term could become more important (with regard to the scaling behaviour) for larger magnetic fields, but this is beyond the field ranges considered in this work.

### Hysteretic behaviour of octupolar ordering

We are motivated in this section by preliminary experimental observations found by some of us (A.S. and S.N., unpublished) of hysteretic behaviour in the length change along the [111] direction below the supposed-octupolar temperature. Hysteresis arises from the existence of domains and the motion of domain walls in the presence of obstructing ‘pinning sites’, which have not been taken into account in the Landau theory we have studied. In order to incorporate such effects, we adapt the phenomenological approach due to Jiles and Atherton^55,56^ which has been used to study hysteresis loops in ferromagnetic and ferroelastic materials. This approach identifies the order parameter (obtained by minimizing the Landau free energy) as its ideal bulk value, where the Landau theory includes a direct coupling *u*_*f*_*mh*^3^ of the ferro-octupolar moment *m* and the external [111] magnetic field. The total macroscopic octupolar moment (*m*_exp_) is obtained by solving the constructed Jiles and Atherton model, which is heuristically derived in Supplementary Note 7. The key point to note is that the hysteresis loop arises from having a time-reversal odd moment (and domains) coupling to the magnetic field.

We present the calculated hysteresis for $T \,<\, T_{\cal{O}}$ in Fig. 2a. The initial condition chosen to obtain the hysteresis loop is such that at *h* = 0, the ideal configuration is not being met (i.e. *m*_exp_ ≠ *m*); this depicts the realistic scenario of having not all domains aligned in the same direction at *h* = 0. The depicted hysteresis is reminiscent of the hysteresis in ferromagnets. We obtain the corresponding length change along the (1, 1, 1) direction as shown in Fig. 2b, which for small magnetic fields displays the linear-in-*h* scaling behaviour. To better observe this linear-in-*h* scaling in the length change, we present the derivative of the length change with respect to the magnetic field in the inset of Fig. 2b. The linear-in-*h* scaling behaviour of the length change is more clearly apparent as a constant *y*-intercept in the inset; the further linear scaling in the inset is due to the background quadratic-in-*h* scaling behaviour of Δ*L*/*L* from the conduction electrons (~*γ* term).

Furthermore, we note that the field strength h* corresponding to the minimum of the length change [i.e. *d*(Δ*L*/*L*)/*dh* = 0] provides a threshold above which the conduction electron background dominates over the linear-in-*h* scaling behaviour. For this particular length change direction, $h^ \ast = \frac{{\sqrt 3 }}{2}\frac{{g_{\cal{O}}|m|}}{{{\gamma} _{\mathrm{c}}}}$. We note that dimensionally *h* has units of energy (as we have set the Bohr magneton, *μ*_B_ = 1 here) and the strain tensor $\epsilon$ is dimensionless; this implies that the composite quantity $g_{\cal{O}}|m|$ is dimensionless, while the conduction electron term *γ*_c_ (which scales like an off-diagonal magnetic susceptibility, from Eq. (6)) has units of (energy)^−1^. Dimensional analysis thus suggests *γ*_c_ ~ DOS, where (DOS) is the conduction electron density of states at the Fermi level. To proceed further with the other quantities, we note that *m* itself is a dimensionless quantity; subsequently $g_{\cal{O}}$ is also dimensionless. This follows from the convention used in Landau theory where $m\sim \left( {\frac{{T_{\cal{O}} - T}}{{T_{\cal{O}}}}} \right)^\beta$, and as such for low temperatures (*T* → 0) we can take *m* as an *O*(1) number. If we also take $g_{\cal{O}}\sim O(1)$, then from above h* ~ DOS^−1^. Thus, the location of the minimum field is inversely dependent on the conduction electron DOS at the Fermi level: when the conduction electron DOS is small, the minimum field h* is correspondingly large.

## Discussion

In this work, motivated by recent and ongoing experiments on Pr(Ti,V,Ir)_2_(Al,Zn)_20_, we have used Landau theory of multipolar orders coupled to lattice strain fields to study magnetostriction in systems with quadrupolar and octupolar orders. Our theoretical results for magnetostriction in the presence of octupolar order appear consistent with recent magnetostriction experiments performed by some of us (A.S. and S.N., unpublished) on PrV_2_Al_20_ where the onset of unusual linear-in-field and hysteretic magnetostriction is observed for fields along the [111] direction for *T* < 0.65 K; in particular, we predict linear-in-*h* scaling of the length change for length changes (both parallel and perpendicular) to magnetic fields applied along the [111] direction, and also for length changes perpendicular to [100], below $T_{\cal{O}}$. Moreover, the coefficient of the linear-in-*h* term is directly proportional to the octupolar moment, thus giving a distinct signature for the onset of octupolar ordering as well as a means to detect/measure the octupolar moment. In addition, we can qualitatively understand the quadratic-in-field background magnetostriction observed in these experiments; we predict that this scaling arises from the quadrupolar moments and/or direct coupling of the conduction electrons to the external magnetic field and the appropriate lattice normal modes. The summary of all the scaling behaviours is presented succinctly in Table 1.

Our results are broadly applicable to a variety of multipolar orders in cubic systems. For instance, the conclusions here are extendable to the cluster octupolar moments suggested in antiferro-magnetically interacting magnetic moments in pyrochlore iridates^57^. Furthermore, the results presented here can be extended to other more ‘typical’ probes of multipolar ordering/fluctuations. For instance, due to the permitted octupolar-strain coupling, we expect to observe elastic constant softening in the elastic constant *c*_44_ at the ordering temperature, $T_{\cal{O}}$, under the application of a magnetic field. We note that it is the *c*_44_ elastic constant that softens, as it is the associated elastic constant with the off-diagonal strain normal modes. Similarly, we expect elasto-resistivity experiments^58^ to be a probe for octupolar susceptibility. The application of an elastic strain with *T*_2*g*_ symmetry (such as *xy*, *xz*, or *yz*) in the presence of a magnetic field would result in an associated anisotropic resistivity (*ρ*_*xy*_, *ρ*_*xz*_, *ρ*_*yz*_), which will be proportional to the octupolar susceptibility. Finally, we expect that Pr(Ti,V,Ir)_2_(Al,Zn)_20_ compounds will possess the characteristic low frequency Raman quasielastic peak^59^, associated with octupolar fluctuations; specifically, under the application of a magnetic field along the [001] direction, we expect the quasielastic peak to appear in the *xy* symmetry Raman spectra.

In terms of future work, an interesting avenue to explore is that of the coupling of the conduction electrons to the multipolar moments, as well as to the lattice strain and magnetic field. In particular, the origin of the conduction electron term in Eq. (6), introduced in our phenomenological model from symmetry arguments, is a fascinating direction to explore (as well as potential other terms arising from conduction electrons). We discuss in Supplementary Note 8 a possible origin of the conduction electron term of Eq. (6). Understanding the nature and role of the conduction electrons will also help shed light on the quantum critical behaviour and superconductivity in such multipolar Kondo lattice systems^4,7,8,32,60–64^.

## Methods

### Non-Kramers ground states of Pr^3+^ ions

The ground states are experimentally found to form a non-Kramers doublet written in |*J*_*z*_〉 basis as

9 $$\begin{array}{l}{\Gamma} _3^{(1)} = \frac{1}{2}\sqrt {\frac{7}{6}} \left| 4 \right\rangle - \frac{1}{2}\sqrt {\frac{5}{3}} \left| 0 \right\rangle + \frac{1}{2}\sqrt {\frac{7}{6}} \left| { \,-\, 4} \right\rangle ,\\ {\Gamma} _3^{(2)} = \frac{1}{{\sqrt 2 }}\left| 2 \right\rangle + \frac{1}{{\sqrt 2 }}\left| { - 2} \right\rangle .\end{array}$$

Constructing a pseudospin basis ({|↑〉, |↓〉}) from the Γ_3*g*_ doublets as

10 $$\begin{array}{l}\left| {\uparrow} \right\rangle = \frac{1}{{\sqrt 2 }}\left[ {\left| {{\Gamma} _3^{(1)}} \right\rangle + i\left| {{\Gamma} _3^{(2)}} \right\rangle } \right],\\ \left| \downarrow \right\rangle = \frac{1}{{\sqrt 2 }}\left[ {i\left| {{\Gamma} _3^{(1)}} \right\rangle + \left| {{\Gamma} _3^{(2)}} \right\rangle } \right]\end{array}$$

allows the multipolar moments to be succinctly written as the effective pseudospin-1/2 operator ***τ*** = (*τ*^*x*^, *τ*^*y*^, *τ*^*z*^) in Eq. (2) in the main text. The local *T*_*d*_ symmetry instilled by the FK cage provides a constraint on the possible terms permitted in the Landau theory. The generating elements of *T*_*d*_ are ${\cal{S}}_{4z}$ (improper rotation of *π*/2 about the $\widehat {\mathbf{z}}$-axis) and ${\cal{C}}_{31}$ (rotation of 2*π*/3 about the body diagonal [111] axis). In addition to these point group symmetries, we also require that the terms in the Landau theory be invariant under spatial inversion about the diamond bond centre ${\cal{I}}$ (which swaps the A and B sublattices), as well as time-reversal Θ. The behaviour of the multipolar moments under these symmetry constraints is detailed in Supplementary Table 1. As described in the main text, we construct our Landau theory using the order parameters *ϕ*, $\tilde \phi$, and *m*.

### Interacting multipolar orders

Equipped with the symmetry knowledge from Supplementary Table 1 we can now write down the Landau free energy for this particular multipolar ordered system as

11 $$F_{{\cal{Q}},{\cal{O}}}[\phi ,\tilde \phi ,m] = F_{\tilde \phi } + F_m + F_\phi + F_{\tilde \phi ,\phi ,m}\;.$$

Here, the free energies $F_{\tilde \phi }$, *F*_*m*_, and *F*_*ϕ*_ denote the independent free energies of the AF${\cal{Q}}$, F${\cal{O}}$, and F${\cal{Q}}$ orders. Setting $\tilde \phi = |\tilde \phi |{\mathrm{e}}^{{\mathrm{i}}\tilde \alpha }$ and *ϕ* = |*ϕ*|e^i*α*^, we get

12 $$F_{\tilde \phi } = \left[ {\frac{{t_{\tilde \phi }}}{2}|\tilde \phi |^2 + u_{\tilde \phi }|\tilde \phi |^4} \right] + \left( {l_{\tilde \phi } + w_{\tilde \phi }\cos (6\tilde \alpha )} \right)|\tilde \phi |^6,$$

13 $$F_m = \left[ {\frac{{t_m}}{2}m^2 + u_mm^4} \right],$$

14 $$F_\phi = \left[ {\frac{{t_\phi }}{2}|\phi |^2 + u_\phi |\phi |^4} \right] + v_\phi \sin (3\alpha )|\phi |^3,$$

The first two terms in Eqs. (12)–(14), in square brackets, are the usual mass and quartic interaction terms for AF${\cal{Q}}$, F${\cal{O}}$ and F${\cal{Q}}$ order parameters. We choose $t_{\tilde \phi } = (T - T_{\cal{Q}})/T_{\cal{Q}}$, and $t_m = (T - T_{\cal{O}}^{(0)})/T_{\cal{O}}^{(0)}$ with $T_{\cal{O}}^{(0)} \,<\, T_{\cal{Q}}$, where *T* denotes the temperature. Focussing on the mass term alone, decreasing *T* will thus lead to an anti-ferroquadrupolar order for *T* < $T_{\cal{Q}}$, and a lower temperature transition into a state with coexisting ferro-octupolar order when $T \,<\, T_{\cal{O}}^{(0)}$. These (bare) transition temperatures will be affected by the interplay of the two order parameters; in particular, the true octupolar transition $T_{\cal{O}}$ will be renormalized from its bare value $T_{\cal{O}}^{(0)}$ due to the onset of quadrupolar order (besides fluctuation effects which we do not consider here). A measure of how close the two transition temperatures are to each other is provided by the ratio ($T_{\cal{Q}}$ − $T_{\cal{O}}$)/($T_{\cal{Q}}$ + $T_{\cal{O}}$). Finally, since F${\cal{Q}}$ is not considered to be a primary order parameter, we choose a large positive mass term, *t*_*ϕ*_. The remaining non-trivial terms in Eqs. (12) and (14) are the unusual sixth order and cubic “clock” terms, with respective coefficients $w_{\tilde \phi }$ and *v*_*ϕ*_, which fix the phases of the AF${\cal{Q}}$ and F${\cal{Q}}$ order parameters. We set $l_{\tilde \phi } \,> \, |w_{\tilde \phi }|$ to ensure that the free energy is bounded from below.

The couplings between the different multipolar order parameters are encapsulated in $F_{\tilde \phi ,\phi ,m}$, namely between AF${\cal{Q}}$ and F${\cal{Q}}$ moments (*g*_1_, *g*_2_), and between the quadrupolar and the octupolar moments $(u_{\phi m},u_{\tilde \phi ,m})$

15 $$F_{\tilde \phi ,\phi ,m} =	 \, \, g_1|\phi ||\tilde \phi |^2\sin (\alpha + 2\tilde \alpha ) + g_2|\tilde \phi |^2|\phi |^2\\ 	+\, u_{\phi m}|\phi |^2m^2 + u_{\tilde \phi m}|\tilde \phi |^2m^2,$$

where the term *g*_1_ is a symmetry-allowed cubic term.

### Coupling of magnetic field to multipolar moments

Due to the lack of magnetic dipole moment supported by the Γ_3*g*_ doublet, the magnetic field does not couple linearly to the states. One can derive the low energy magnetic field Hamiltonian by performing second-order perturbation theory in **h** ⋅ **J**, where the low energy subspace is spanned by the Γ_3*g*_ doublet, and the high energy subspace is spanned by the excited triplets Γ_4,5_; here **h** has units of energy as we have set the Bohr magneton, *μ*_B_ = 1. This leads to

16 $${\cal{H}}_{{\mathrm{eff}}} =	 {\gamma} _0\left[ {\frac{{\sqrt 3 }}{2}(h_x^2 - h_y^2)\tau ^x + \frac{1}{2}(3h_z^2 - h^2)\tau ^y} \right]\\ =	 \psi _H^ \ast \tau ^ + + \psi _H\tau ^ - \sim \psi _H^ \ast \phi + \psi _H\phi ^ \ast \;.$$

In the above Eq. (16), **h** = (*h*_*x*_, *h*_*y*_, *h*_*z*_) with |**h**| = *h*, and ${\gamma} _0 \equiv \frac{{ - 14}}{{3\Delta ({\Gamma} _4)}} + \frac{2}{{\Delta ({\Gamma} _5)}}$, where Δ(Γ_4_), Δ(Γ_5_) are the gaps between the low energy doublets and the corresponding triplet states at zero magnetic field. The effective coupling to the ferroquadrupolar order is via $\psi _H \equiv \frac{{{\gamma} _0\sqrt 3 }}{4}(h_x^2 - h_y^2) + {\mathrm{i}}\frac{{{\gamma} _0}}{4}(3h_z^2 - h^2)$. Based on the form of the coupling in Eq. (16), we infer that *ψ*_*H*_ transforms identically to *ϕ* under the relevant symmetries. Going to third-order in perturbation theory leads to a further ${\cal{O}}$(*h*^3^) coupling of the magnetic field to octupole moment of the form ~*h*_*x*_*h*_*y*_*h*_*z*_*τ*^*z*^.

Thus, the symmetry-allowed effective magnetic field coupling to the quadrupolar moments is

17 $$\begin{array}{l}F_{{\mathrm{mag}}}[\phi ,\tilde \phi ] = \tilde r_H\sin (\theta _H + 2\tilde \alpha )|\tilde \phi |^2|\psi _H|\\ \hskip 41pt +\, r_H\cos (\alpha - \theta _H)|\phi ||\psi _H|\\ \hskip 41pt +\, \left( {\tilde s_H|\tilde \phi |^2 + s_H|\phi |^2} \right)h^2,\end{array}$$

where $|\psi _H| = \frac{{{\gamma} _0}}{4}\sqrt {3(h_x^2 - h_y^2)^2 + (3h_z^2 - h^2)^2}$, and $\tan (\theta _H) = \frac{1}{{\sqrt 3 }}\frac{{3h_z^2 - h^2}}{{(h_x^2 - h_y^2)}}$. The first (second) line in Eq. (17) is the symmetry-allowed coupling to the AF${\cal{Q}}$ (F${\cal{Q}}$). The third line involves couplings permitted due to pure symmetry reasons that renormalize the mass terms of the AF${\cal{Q}}$ and F${\cal{Q}}$. Physically they arise from conduction electron mediated magnetic couplings (having integrated out the conduction electrons); similar coupling to the octupolar moment is also permitted [~*h*^2^*m*^2^], which is formally introduced via the magnetic field assisted coupling of the octupolar moment to the lattice strain. In the main text, we discuss magnetic fields applied along the [100], [110] and [111] directions. For clarity, we present the value for |*ψ*_*H*_| and *θ*_*H*_ for the magnetic field directions discussed in subsequent sections in Table 2. In the presence of the magnetic field, it is possible for additional couplings between the quadrupolar and octupolar moments to be induced, such as

18 $$\sim m^2\cos (\tilde \alpha - \theta _H)|\tilde \phi ||\psi _H|\;,$$

19 $$\sim m^2\sin (\theta _H + 2\tilde \alpha )|\tilde \phi |^2|\psi _H|\;,$$

20 $$\sim m^2|\phi |^2\left( {h_x^2 + h_y^2 + h_z^2} \right)\;,$$

21 $$\sim m^2|\tilde \phi |^2\left( {h_x^2 + h_y^2 + h_z^2} \right)\;.$$

These terms are merely the usual quadratic-in-field coupling to the quadrupolar moment (Eqs. (16) and (17)) with *m*^2^ multiplied into it. Due to symmetry constraints, we cannot have terms which are linear in the octupolar, quadrupolar and magnetic field (breaks ${\cal{C}}_{31}$ symmetry). These above terms do not affect the leading scaling behaviour of the magnetostriction, as they have the same order of *h* as previous terms in the free energy. Specifically, the terms are quadratic-in-*h* and can be thought of as renormalizing the mass term of the octupolar moment. We recall that the octupolar mass term already contains a quadratic-in-*h* term, which arose from integrating out the elastic strain in Eq. (6), and so these new terms merely modify the coefficient of the previous quadratic-in-*h* expressions/terms.

**Table 2:** Effective magnetic field strengths |*ψ*_*H*_| and associated complex angle *θ*_*H*_

Magnetic field, h = *h* $\widehat {\mathbf{n}}$	|*ψ*_*H*_|	*θ*_*H*_
$\widehat {\mathbf{n}}$ = [100]	$\frac{{\gamma _0}}{2}h^2$	−*π*/6
$\widehat {\mathbf{n}} = \frac{1}{{\sqrt 2 }}[110]$	$\frac{{\gamma _0}}{4}h^2$	−*π*/2
$\widehat {\mathbf{n}} = \frac{1}{{\sqrt 3 }}[111]_{}^{}$	0	–

For the $\widehat {\mathbf{n}} = \frac{1}{{\sqrt 3 }}[111]$, the magnetic field does not directly couple to the quadrupolar moments, but can do so via $\tilde s_H$ and *s*_*H*_.

The Landau theory is numerically minimized using standard minimization/optimization schemes. The hysteresis differential equation is numerically solved using Runge-Kutta 4th order methods. We use the initial condition of *m*_ir_ = 0 for *h* = 0 to obtain the depicted solution, with *k* = 100, *α* = 10^−3^, *c* = 0.01.

## Supplementary information

Supplementary Information

## Data Availability

All relevant data are available upon reasonable request to the corresponding author.

## Code availability

All relevant codes are available upon reasonable request to the corresponding author.
