# Supplementary material for: Unveiling hidden multipolar orders with magnetostriction
Source: Nat Commun. 2019 Sep 9;10:4092. doi: 10.1038/s41467-019-11913-3 (PMC6733943; doi:10.1038/s41467-019-11913-3)
Supplement: Supplementary file 1 — Supplementary Information [file 41467_2019_11913_MOESM1_ESM.pdf]

Supplementary Information: Unveiling Hidden Multipolar Orders with Magnetostriction

Adarsh S. Patri,[1] Akito Sakai,[2,3] SungBin Lee,[4] Arun Paramekanti,[1] Satoru Nakatsuji,[2,3] and Yong Baek Kim[1]

[1]*Department of Physics and Centre for Quantum Materials, University of Toronto, Toronto, Ontario M5S 1A7, Canada*

[2]*Institute for Solid State Physics, University of Tokyo, Kashiwa, Chiba 277-8581, Japan*

[3]*CREST, Japan Science and Technology Agency (JST), 4-1-8 Honcho Kawaguchi, Saitama 332-0012, Japan*

[4]*Department of Physics, Korea Advanced Institute of Science and Technology, Daejeon, 34141, Korea*

SUPPLEMENTARY NOTE 1: GENERAL EXPRESSION OF LENGTH CHANGE IN DIFFERENT DIRECTIONS

The relative length change, $\Delta L/L$, of the crystal can be shown to be related to the components of the strain tensor. To derive such an expression we consider a pair of neighbouring points (A and B) situated at $\mathbf{x}$ and $\mathbf{x}+\Delta\mathbf{x}$, respectively, in the undeformed crystal. The vector separating these two points is $\Delta\mathbf{x} = \Delta s\hat{\boldsymbol{\ell}}$, where Δs is the distance between A and B, and $\hat{\boldsymbol{\ell}} \equiv \mathbf{e}_{\mathrm{A}\to\mathrm{B}}$ is the unit vector directed from A to B. Under a deformation, the points A and B get respectively shifted by displacement vectors $\mathbf{u}$ and $\mathbf{u}(\mathbf{x}+\Delta\mathbf{x})$ to new locations $\mathbf{y}(\mathbf{x}) = \mathbf{x} + \mathbf{u}(\mathbf{x})$ and $\mathbf{y}(\mathbf{x}+\Delta\mathbf{x}) = [\mathbf{x}+\Delta\mathbf{x}] + \mathbf{u}(\mathbf{x}+\Delta\mathbf{x})$. The relative vector connecting these neighbouring points in the deformed crystal is $\Delta\mathbf{y} = \Delta\mathbf{x} + \Delta\mathbf{u}$, where for small enough displacements $\Delta u_i = \Sigma_k \frac{\partial u_i}{\partial x_k}\Delta x_k$; $i = 1,2,3$ is the unit vector directions, and k is being summed over. Expanding the relative length change ($|\Delta\mathbf{y}| - |\Delta\mathbf{x}|$) over the initial separation of the points ($|\Delta\mathbf{x}|$) leads to the general expression of the length change along a direction $\boldsymbol{\ell}$:

$$\left(\frac{\Delta L}{L}\right)_{\boldsymbol{\ell}} = \sum_{i,j=1}^{3} \epsilon_{ij}\hat{\ell}_i\hat{\ell}_j \ , \tag{1}$$

where $\epsilon_{ij} \equiv \frac{1}{2}\left(\frac{\partial u_i}{\partial x_j} + \frac{\partial u_j}{\partial x_i}\right)$ is the familiar strain tensor, and $\hat{\ell}_i$ is the ith component of the unit vector $\hat{\boldsymbol{\ell}}$.

SUPPLEMENTARY NOTE 2: SYMMETRY TRANSFORMATIONS OF MULTIPOLAR ORDER PARAMETERS

Under the symmetry constraints detailed in the main text, the multipolar moments transform as denoted in Supplementary Table 1. In real $\mathbb{R}^3$ space, the matrix representations of $\mathcal{S}_{4z}$ and $\mathcal{C}_{31}$ are,

$$\mathcal{S}_{4z} = \begin{bmatrix} 0 & -1 & 0 \\ 1 & 0 & 0 \\ 0 & 0 & 1 \end{bmatrix} \cdot \mathbb{I}, \qquad \mathcal{C}_{31} = \begin{bmatrix} 0 & 0 & 1 \\ 1 & 0 & 0 \\ 0 & 1 & 0 \end{bmatrix},$$

where $\mathbb{I}$ denotes parity $(x,y,z) \to (-x,-y,-z)$.

Symmetry	F$\mathcal{Q}$	F$\mathcal{O}$	AF$\mathcal{Q}$	AF$\mathcal{O}$
$\mathcal{I}$	$\phi \to \phi$	$m \to m$	$\tilde{\phi} \to -\tilde{\phi}$	$\tilde{m} \to -\tilde{m}$
Θ	$\phi \to \phi^*$	$m \to -m$	$\tilde{\phi} \to \tilde{\phi}^*$	$\tilde{m} \to -\tilde{m}$
$\mathcal{S}_{4z}$	$\phi \to -\phi^*$	$m \to -m$	$\tilde{\phi} \to -\tilde{\phi}^*$	$\tilde{m} \to -\tilde{m}$
$\mathcal{C}_{31}$	$\phi \to \mathrm{e}^{-\mathrm{i}\frac{2\pi}{3}}\phi$	$m \to m$	$\tilde{\phi} \to \mathrm{e}^{-\mathrm{i}\frac{2\pi}{3}}\tilde{\phi}$	$\tilde{m} \to \tilde{m}$

Supplementary Table 1. Transformation of multipolar order parameters under generating elements of T_d ($\mathcal{S}_{4z}$, $\mathcal{C}_{31}$), bond centre inversion ($\mathcal{I}$) and time reversal (Θ). The '$*$' indicates complex conjugation.

SUPPLEMENTARY NOTE 3: VALUES OF LANDAU PARAMETERS

The values of the Landau parameters are arbitrary to a certain extent, and depending on the choice of the parameters the subsequent scaling coefficients are altered. For the studies in this work, we used the following values for the Landau parameters (in the appropriate units): $T^{\mathrm{c}}_{\mathcal{Q}} = 40$ and $T^{\mathrm{c}}_{\mathcal{O}} = 6$, $u_{\tilde{\phi}} = u_m = u_\phi = w_{\tilde{\phi}} = -u_{\tilde{\phi}m} = -u_{\phi m} = 5$, $l_{\tilde{\phi}} = 6$, $v_\phi = -2$, $\tilde{r}_H = r_H = 0.05$, $\tilde{s}_H = s_H = -0.0006$, $(g_1, g_2) = (-1.7, 3.7)$, $g_{\mathcal{O}} = 2.19$, $g_{\mathcal{Q}} = 0.4$, $(c_{11} - c_{12}) = 10^2$, and $c_{44} = 2.4\times10^2$. The values for the elastic modulus tensor components are chosen to be large, as they are only responsible for shifting the critical temperatures (the mass term) of AF$\mathcal{Q}$, F$\mathcal{O}$ (F$\mathcal{Q}$).

SUPPLEMENTARY NOTE 4: MULTIPOLAR ORDER PARAMETERS WITH RESPECT TO MAGNETIC FIELD STRENGTH

Supplementary Figures 1, 2, 3 presents the solutions of the order parameters from a thorough numerical study of the complete Landau free energy. As can be seen, the F$\mathcal{Q}$ moment is indeed an even function-in-h. Prior to discussing the particular magnetic field directions, we examine the consequences of our choice of parameters, which have the following properties

$$v_\phi < 0, \quad w_{\tilde{\phi}} > 0, \quad g_1 < 0, \quad \tilde{r}_H > 0, \quad r_H > 0. \tag{2}$$

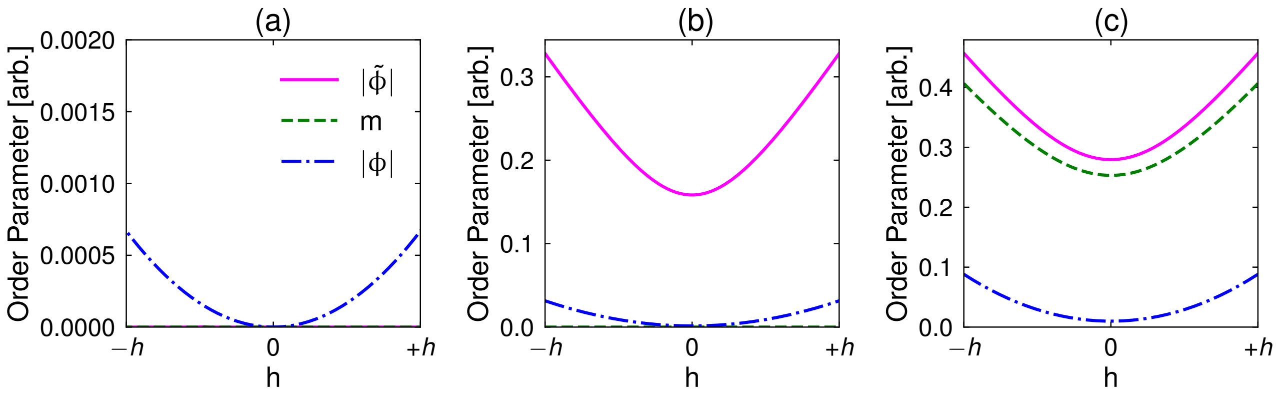

Supplementary Figure 1. Order parameters AF$\mathcal{Q}$ [$|\tilde{\phi}|$], F$\mathcal{O}$ [m], F$\mathcal{Q}$ [$|\phi|$] versus magnetic field strength h applied along [100] direction, for (a) $T > T_{\mathcal{Q}}, T_{\mathcal{O}}$, (b) $T_{\mathcal{O}} < T < T_{\mathcal{Q}}$, and (c) $T < T_{\mathcal{Q}}, T_{\mathcal{O}}$. Qualitatively, the multipolar moments possess even-in-h symmetry.

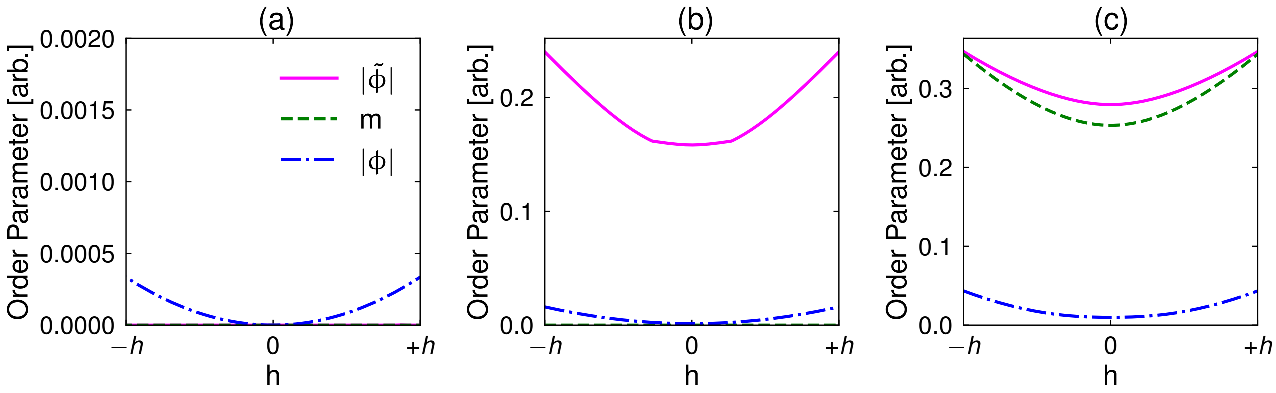

Supplementary Figure 2. Order parameters AF$\mathcal{Q}$ [$|\tilde{\phi}|$], F$\mathcal{O}$ [m], F$\mathcal{Q}$ [$|\phi|$] versus magnetic field strength h applied along [110] direction, for $T > T_{\mathcal{Q}}, T_{\mathcal{O}}$, (b) $T_{\mathcal{O}} < T < T_{\mathcal{Q}}$, and (c) $T < T_{\mathcal{Q}}, T_{\mathcal{O}}$. Qualitatively, the multipolar moments possess even-in-h symmetry.

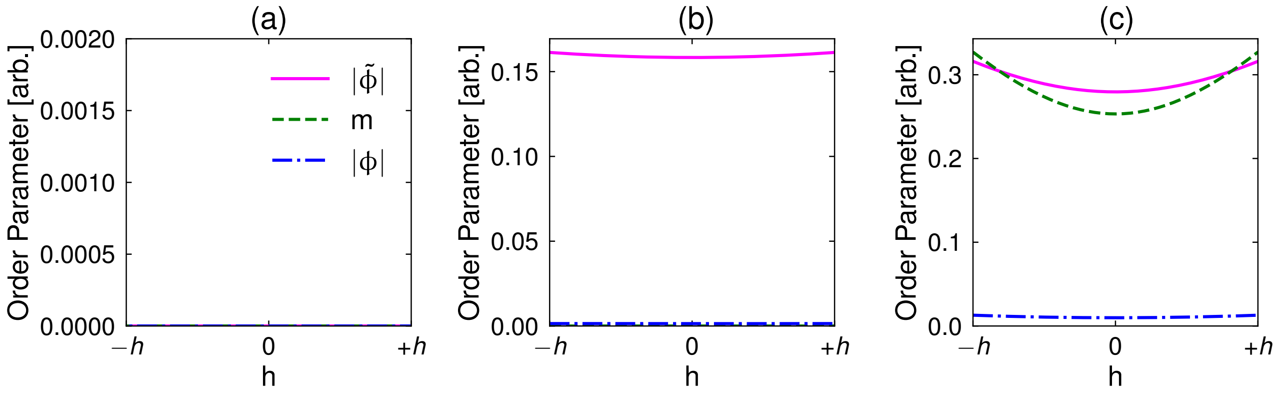

Supplementary Figure 3. Order parameters AF$\mathcal{Q}$ [$|\tilde{\phi}|$], F$\mathcal{O}$ [m], F$\mathcal{Q}$ [$|\phi|$] versus magnetic field strength h applied along [111] direction, for (a) $T > T_{\mathcal{Q}}, T_{\mathcal{O}}$, (b) $T_{\mathcal{O}} < T < T_{\mathcal{Q}}$, and (c) $T < T_{\mathcal{Q}}, T_{\mathcal{O}}$. Qualitatively, the multipolar moments possess even-in-h symmetry.

In minimizing the free energy, we examine each of the complex angular-dependent terms and determine the value of the corresponding angles that minimize (magnetic field independent terms in) the Landau free energy

$$\begin{aligned} &|w_{\tilde{\phi}}||\tilde{\phi}|^6\cos(6\tilde{\alpha}) && \overset{\min[\tilde{\alpha}]}{\Longrightarrow} \tilde{\alpha}=\frac{\pm\pi}{6},\frac{\pm\pi}{2},\frac{\pm 5\pi}{6} \\ &-|v_{\phi}||\phi|^3\sin(3\alpha) && \overset{\max[\alpha]}{\Longrightarrow} \alpha=\frac{-\pi}{2},\frac{\pi}{6},\frac{5\pi}{6} \\ &-|g_1||\phi||\tilde{\phi}|^2\sin(\alpha+2\tilde{\alpha}) && \overset{\max[\alpha,\tilde{\alpha}]}{\Longrightarrow} (\alpha,\tilde{\alpha})=\left(\frac{5\pi}{6},\frac{5\pi}{6}\right) \end{aligned} \tag{3}$$

In the absence of external magnetic fields, there is a built-in degeneracy that allows a host of possible solutions, namely

$$\begin{aligned} (\alpha,\tilde{\alpha}) =&(-\pi/2,\pm\pi/2),(\pi/6,-5\pi/6),(\pi/6,\pi/6), \\ &(5\pi/6,-\pi/6),(5\pi/6,5\pi/6)\ . \end{aligned} \tag{4}$$

$\mathbf{h} \parallel [100]$

For this magnetic field direction, we now examine the magnetic field dependent complex angular terms and determine the value of the corresponding angles that minimize the Landau free energy (having set $\theta_H = -\pi/6$),

$$\begin{aligned} &|r_H|\cos(\alpha-\theta_H)|\psi_H||\phi| && \overset{\min[\alpha]}{\Longrightarrow} \tilde{\alpha}=\frac{-7\pi}{6},\frac{5\pi}{6} \\ &|\tilde{r}_H||\psi_H||\tilde{\phi}|^2\sin(-\frac{\pi}{6}+2\alpha) && \overset{\min[\tilde{\alpha}]}{\Longrightarrow} \alpha=\frac{-\pi}{6},\frac{5\pi}{6} \end{aligned} \tag{5}$$

Thus, the choice of $(\alpha,\tilde{\alpha}) = (\frac{5\pi}{6},\frac{5\pi}{6})$ extremizes all of the above expressions simultaneously. This result is the justification of the zero-field-limit conclusion stated above, where the $h = 0$ limit is achieved by tuning down a magnetic field applied along the [100] direction such that the $\alpha = 5\pi/6$ solution is chosen. Now, the scaling behaviour of the quadrupole moment can be understood by deriving approximate analytical expressions; this is easiest to calculate for $T > T_{\mathcal{Q}}, T_{\mathcal{O}}$, and more tedious in the other two temperature regimes. The numerical solution of the order parameters in the three temperature regimes is presented in Supplementary Fig. 1.

For $T > T_{\mathcal{Q}}, T_{\mathcal{O}}$, $|\tilde{\phi}|$ and m are zero. Extremizing the complete free eenergy with $\tilde{\phi} = m = 0$ [and knowledge of $\alpha = 5\pi/6$], and reasonably assuming that parasitic ϕ is small, we arrive at

$$|\phi|\approx\left(\frac{\gamma_0}{2}|r_H|\right)h^2\ , \tag{6}$$

where we have reintroduced $|\psi_H| = \frac{\gamma_0}{2}h^2$.

A similar (albeit more tedious) approach can be adapted to determine an approximate scaling behaviour in the other two temperature regimes of $T_{\mathcal{O}} < T < T_{\mathcal{Q}}$ (where $|\phi|, |\tilde{\phi}| \neq 0$ and $m = 0$) and $T < T_{\mathcal{Q}}, T_{\mathcal{O}}$ (where all of $|\phi|, |\tilde{\phi}|, m \neq 0$). Doing so, we arrive at the leading-order-in-h scaling of $|\phi| \approx \big|_{T<T_{\mathcal{Q}}}\phi_0 + \phi_h h^2$, where $\phi_{0,h}$ are a collection of constants. The constant shift ϕ_0 result is reasonable as below $T_{\mathcal{Q}}$ the AF$\mathcal{Q}$ is non-vanishing even at zero magnetic field, and thus the accompanying parasitic F$\mathcal{Q}$ is finite for zero magnetic field. We affirm these scaling behaviours by performing a thorough numerical study of the full Landau free energy in the temperature regimes of interest in Supplementary Fig. 1. As can be seen in Supplementary Fig. 1, the F$\mathcal{Q}$ moment is indeed an even function-in-h. Moreover, for $T < T_{\mathcal{Q}}$, the F$\mathcal{Q}$ moment is finite even at zero magnetic field due to the AF$\mathcal{Q}$ having spontaneously ordered.

$\mathbf{h} \parallel [110]$

For magnetic fields along this direction, unlike the [110] direction, there is a lack of harmony in the complex angles that minimize the Landau free energy. For the AF$\mathcal{Q}$ and F$\mathcal{Q}$ moments, the complex angles that extremize the respective magnetic field dependent terms are

$$\begin{aligned} &|\tilde{r}_H|\sin(\frac{-\pi}{2}+2\tilde{\alpha})|\psi_H||\tilde{\phi}|^2 && \overset{\min[\tilde{\alpha}]}{\Longrightarrow} \tilde{\alpha}=0,\pm\pi \\ &|r_H|\cos(\alpha-(-\frac{\pi}{2}))|\psi_H||\phi| && \overset{\min[\alpha]}{\Longrightarrow} \alpha=\frac{\pi}{2} \end{aligned} \tag{7}$$

This lack of harmony in the choice of polar angle leads to a competition between the two terms: the magnetic field dependent terms desire $\tilde{\alpha} = 0$ and $\alpha = \pi/2$; while we recall from Eq. 4 the anisotropic sextic (cubic) term desires $\tilde{\alpha} = \pm 5\pi/6$ ($\alpha = 5\pi/6$) [amongst other angles; not the same as the ones preferred by this magnetic field]. Thus a simple analytical solution for the scaling behaviour of the relative length change is not as easy to derive (nor illuminating). Nevertheless, just as for $\mathbf{h} \parallel [100]$, the quadrupolar order parameters are even-functions-in-h as can be seen in Supplementary Fig. 2 and corroborated by numerical fits of the order parameters.

We stress on the atypical nature of the [110] direction, in that it introduces a degeneracy into the system. We can observe this by just focusing on the AF$\mathcal{Q}$ moment's terms, where if we take

$$\begin{aligned} \tilde{\alpha}&=0+x && \Longrightarrow \cos(6\tilde{\alpha})=\cos(6x)\ , \\ \tilde{\alpha}&=0+x && \Longrightarrow \sin(-\pi/2+2\tilde{\alpha})=-\cos(2x)\ , \\ \alpha&=\pi/2+y && \Longrightarrow \sin(3\tilde{\alpha})=-\cos(3y)\ , \\ \alpha&=\pi/2+y && \Longrightarrow \cos(\alpha-(-\pi/2))=-\cos(y)\ , \end{aligned} \tag{8}$$

where x and y are the deviations from 0 and $\pi/2$, respectively. These terms are invariant under $x \to -x$ and $y \to -y$, resulting in two equivalent orientations for the quadrupolar moment. It is this symmetry that is responsible for degeneracy and the observed 'multiple' solutions. The 'multiple' solutions persist for small magnetic field strengths (where the competition with the anisotropic term is still ongoing); however, for large enough field strengths, the magnetic field term dominates over the anisotropic term to yield one unique solution determined

by the magnetic field term. The point at which the magnetic field has dominated and takes over the anisotropic term is observed as a 'kink' in the order parameters, as seen in Supplementary Fig. 2; above this h_{kink} the 'multiple' solutions vanishes, and a single/unique solution emerges. Note that this 'multiple' solutions phenomena is not unique to the [110] field direction; it can readily be transferred over to the [100] direction's solutions by flipping the sign of the chosen parameters $v_\phi, \tilde{r}_g, g_1$, which then gives the pure/unique solution to [110]. Nevertheless, this degeneracy does not impact the observed scaling (even-in-h) behaviour.

In temperature regimes of $T < T_{\mathcal{Q}}$, there is the aforementioned kink at h_{kink} in both the AF$\mathcal{Q}$ and F$\mathcal{Q}$ order parameters for $T_{\mathcal{O}} < T < T_{\mathcal{Q}}$ and $T < T_{\mathcal{Q}}, T_{\mathcal{O}}$, respectively, as seen in Supplementary Figures 2(b,c); the kink is more noticeable in the $|\tilde{\phi}|$ than $|\phi|$ due to the small, parasitic nature of F$\mathcal{Q}$. It is interesting to note that this 'kink' appears for a larger value of h for $T < T_{\mathcal{Q}}, T_{\mathcal{O}}$ than in $T_{\mathcal{O}} < T < T_{\mathcal{Q}}$. This stems from the fact that for T much lower than $T_{\mathcal{Q}}$, the $|\tilde{\phi}|^6$ and $|\phi|^3$ contribution to the free energy is substantial, and thus to overcome these anisotropic terms it requires a larger magnetic field. Hence, the value of h_{kink} grows the lower in T we go (below $T_{\mathcal{Q}}$). Apart from this 'kink', the scaling relations of the F$\mathcal{Q}$ is as expected with a constant zero-in-h shift being present for $T < T_{\mathcal{Q}}$, due to presence of spontaneously ordered AF$\mathcal{Q}$. We also note that for large enough h, the AF$\mathcal{Q}$ is tuned to zero with the ferro-like moments surviving.

$\mathbf{h} \parallel [111]$

For magnetic fields applied along this direction, all the multipolar moments couple quadratically to the magnetic field. We first discuss the magnetic field dependency of the quadrupolar moments. For $T > T_{\mathcal{Q}}$, both quadrupolar moments are zero [as depicted in the numerical solution Supplementary Fig. 3(a)] due to AF$\mathcal{Q}$ not spontaneously ordering, and the lack of a linear-in-$|\phi|$ coupling to the magnetic field for the F$\mathcal{Q}$ moment. For $T < T_{\mathcal{Q}}$, the AF$\mathcal{Q}$ has spontaneously ordered, and hence also permits a finite (but small) parasitic F$\mathcal{Q}$ moment, with a weak quadratic-in-h scaling for F$\mathcal{Q}$; thus $|\phi| \approx \big|_{T_{\mathcal{O}}<T<T_{\mathcal{Q}}} \phi_0 + \phi_h h^2$ here, as seen in Supplementary Fig. 3(b).

The quadratic-in-h scaling of the octupolar moment can be observed by performing a simple analytical approximation, where for the sake of simplicity, we assume only pure F$\mathcal{O}$ ordering (setting the quadrupolar moments to zero). This simplified Landau free energy is of the form,

$$F[m] = \left[\frac{t_m}{2} - \frac{g_{\mathcal{O}}^2}{2c_{44}}(h^2)\right] m^2 + u_m m^4 \ , \tag{9}$$

where we have taken $h_x = h_y = h_z = h/\sqrt{3}$. Extremizing this free energy, and assuming T is far enough below the octupolar critical temperature such that $\frac{g_{\mathcal{O}}^2 h^2/c_{44}}{|t_m|}$ is small enough to Taylor-expand to yield

$$m \approx \sqrt{\frac{|t_m|}{4u_m}} \left(1 + \frac{g_{\mathcal{O}}^2 h^2}{2c_{44}|t_m|}\right) \ . \tag{10}$$

Thus substituting this (simple) approximate solution into Eq. 11 yields a linear in h behaviour in the length change (superimposed on a quadratic background). In the presence of all order parameters, we obtain (from thorough numerical minimization of the full Landau free energy) the same scaling relation of the octupolar (and quadrupolar) moments i.e. $m \sim a_0 + a_2 h^2$, where $a_{0,2}$ are finite constants. The even-in-h scaling behaviour of the multipolar moments is apparent qualitatively in Supplementary Fig. 3(c). We note that the F$\mathcal{Q}$ moment in Supplementary Fig. 3(b) is small but finite as it physically corresponds to an induced moment (i.e. with a large positive mass term). This 'smallness' at $h = 0$ for the AF$\mathcal{Q}$ moment (and subsequently F$\mathcal{Q}$ moment as $|\phi| \sim \sqrt{|\tilde{\phi}|}$ at leading order) is then reflected over the finite magnetic field sweep of Supplementary Fig. 3(b) due to the quadrupolar moment not coupling directly (from second-order perturbation theory in $\mathbf{h} \cdot \mathbf{J}$) to the magnetic field for this field direction. In Supplementary Fig. 3(c), the F$\mathcal{Q}$ is more appreciable, as the AF$\mathcal{Q}$ moment is larger as well since we are lower in temperature.

It is important to note that this scaling behaviour was performed under the assumption that we could neglect the $\mathcal{O}(h^3)$ coupling between the octupolar moment and the magnetic field due to the weak, perturbative field strength we consider in this study i.e. neglected $h_x h_y h_z m$ in the Landau free energy. For magnetic fields along [100] and [110] this term is zero, and plays no role anyway, but for magnetic fields along the [111] direction it is non-zero (albeit small relative to the $\sim h^2 m^2$ coupling).

SUPPLEMENTARY NOTE 5: ESTIMATION OF COUPLING CONSTANTS $g_{\mathcal{O}}$, γ

We can employ the expressions in Eq. 11 in conjunction with experimental measurements to obtain the values of some of the phenomenological coupling constants such as $g_{\mathcal{O}}$ and γ.

Firstly, we can obtain a lower bound for $g_{\mathcal{O}}$. As an example, one can take the length change along (0,1,1) and magnetic field along [100]. The linear-in-h coefficient can be experimentally acquired from magnetostriction measurements; let us denote this coefficient as [experimentally-measured linear-in-h coeff.]. Theoretically, from Eq. 11, we predict this coefficient is equal to $\frac{g_{\mathcal{O}} m}{c_{44}}$. Since m is a dimensionless Landau order parameter near the critical point, we can reasonably take it to be smaller than 1. The value of c_{44} can be obtained for PrV_2Al_{20} from ultrasound measurements[1]. Putting these together, we thus have

a lower bound on the coupling constant i.e. $g_{\mathcal{O}} \geq$ [experimentally-measured linear-in-h coeff.]c_{44}.

Secondly, the value γ can directly be inferred by performing various length change experiments under the magnetic fields proposed in Table 1 in the main text. For instance, obtaining the coefficient of the quadratic-in-h scaling behaviour for length changes along (1,1,1) direction with a parallel magnetic field would directly provide γ. Similarly, the length change along the (1,-1,1) direction for magnetic field along [110] is solely dependent on γ, and such provides a direct means to determine its value.

SUPPLEMENTARY NOTE 6: COMPLETE GENERALIZED LENGTH CHANGE EXPRESSIONS

In this section, we detail the generalized length change expressions along the various high-symmetry directions of the crystal, subject to magnetic fields (parallel and/or perpendicular). For each magnetic field direction, we examine three temperature regimes, namely: above all critical temperatures, between the quadrupolar and octupolar critical temperatures, and below both critical temperatures.

$$\left(\frac{\Delta L}{L}\right)_{(1,0,0)} = \frac{\epsilon_{\rm B}}{3} - \frac{\epsilon_\nu}{2\sqrt{3}} + \frac{\epsilon_\mu}{2} = \frac{1}{3}\epsilon_{\rm B} + \left[-g_{\mathcal{Q}}\frac{\sin(\alpha)-\sqrt{3}\cos(\alpha)}{2\sqrt{3}(c_{11}-c_{12})}\right]|\phi| \,,$$

$$\left(\frac{\Delta L}{L}\right)_{(0,1,\pm1)} = \frac{\epsilon_{\rm B}}{3} + \frac{\epsilon_\nu}{4\sqrt{3}} - \frac{\epsilon_\mu}{4} \pm \epsilon_{yz} = \frac{1}{3}\epsilon_{\rm B} + \left[g_{\mathcal{Q}}\frac{\sin(\alpha)-\sqrt{3}\cos(\alpha)}{4\sqrt{3}(c_{11}-c_{12})}\right]|\phi| \pm \frac{g_{\mathcal{O}} m}{c_{44}} h_x \pm \frac{\gamma_{\rm c} h_y h_z}{c_{44}} \,,$$

$$\left(\frac{\Delta L}{L}\right)_{(1,\pm1,0)} = \frac{\epsilon_{\rm B}}{3} - \frac{\epsilon_\nu}{2\sqrt{3}} \pm \epsilon_{xy} = \frac{1}{3}\epsilon_{\rm B} + \left[-g_{\mathcal{Q}}\frac{\sin(\alpha)}{2\sqrt{3}(c_{11}-c_{12})}\right]|\phi| \pm \frac{g_{\mathcal{O}} m}{c_{44}} h_z \pm \frac{\gamma_{\rm c} h_x h_y}{c_{44}} \,,$$

$$\left(\frac{\Delta L}{L}\right)_{(1,\pm1,1)} = \frac{\epsilon_{\rm B}}{3} + \frac{2\left(\pm\epsilon_{xy} \pm \epsilon_{yz} + \epsilon_{xz}\right)}{3} = \frac{1}{3}\epsilon_{\rm B} + \frac{2g_{\mathcal{O}} m}{3c_{44}}\left[\pm h_z \pm h_x + h_y\right] + \frac{2\gamma_{\rm c}}{3c_{44}}\left[\pm h_x h_y \pm h_y h_z + h_x h_z\right] ,$$

$$\left(\frac{\Delta L}{L}\right)_{(\mp1,1,\pm2)} = \frac{\epsilon_{\rm B}}{3} + \frac{\epsilon_\nu}{2\sqrt{3}} + \frac{\left(\mp\epsilon_{xy} \pm 2\epsilon_{yz} - 2\epsilon_{xz}\right)}{3} = \frac{1}{3}\epsilon_{\rm B} + \left[g_{\mathcal{Q}}\frac{\sin(\alpha)}{2\sqrt{3}(c_{11}-c_{12})}\right]|\phi| + \frac{g_{\mathcal{O}} m}{3c_{44}}\left[\mp h_z \pm 2h_x - 2h_y\right] + \frac{\gamma_{\rm c}}{3c_{44}}\left[\mp h_x h_y \pm 2h_y h_z - 2h_x h_z\right] \,. \tag{11}$$

Here we have used the definition of the normal modes in the main text, and define $\overline{g_{\mathcal{Q}}} \equiv \frac{g_{\mathcal{Q}}}{\sqrt{3}(c_{11}-c_{12})}(...)$, wherein (...) includes the complex-angle dependent terms in Eq. 11. The exact form of which complex angle term is included (1): $\frac{\sin(\alpha)-\sqrt{3}\cos(\alpha)}{2\sqrt{3}(c_{11}-c_{12})}$ or (2): $\frac{\sin(\alpha)}{2\sqrt{3}(c_{11}-c_{12})}$ can be inferred from context i.e. the direction of length change examined $\boldsymbol{\ell}$ under particular magnetic field direction $\hat{\mathbf{n}}$. We use $\Phi_{1,2}$ and $\kappa_{1,2}$ to denote these two scenarios in the main text, when the quadrupolar moment is expanded up to quadratically-in-h; the subscripts 1,2 label the two possible complex angular dependencies in $\overline{g_{\mathcal{Q}}}$ presented above. The key point to retain is that the parasitic F$\mathcal{Q}$ moment is small as compared to the conduction electron generated terms ($\sim \gamma_{\rm c}$). Equation 11 can be used to study the length changes with respect to any magnetic field directions upon appropriate choice of $\mathbf{h} = (h_x, h_y, h_z)$.

SUPPLEMENTARY NOTE 7: DOMAIN WALL MODEL OF OCTUPOLAR MOMENTS

We provide a more detailed derivation of the hysteresis model of octupolar moments. The derivation follows that of Jiles and Atherton[2] with a few modifications that we elaborate on.

The basic premise is that of two equally large (in terms of volume) domains of octupolar order separated by a domain wall. This domain wall is assumed to lie directly on top of a so-called pinning site. A pinning site can be any object that obstructs the motion of domain walls under the influence of a magnetic field; the true nature of the pinning site is not of great importance to the derivation presented here. The domains possess an octupolar moment per unit volume, $m_{\rm d}$. Due to the Ising-like nature of the octupolar moment, one can think of one domain being $m_{\rm d} = +m_{\rm d}$, while the other domain being $m'_{\rm d} = -m_{\rm d}$. The interaction of each domain's octupolar moment with the magnetic field and the bulk octupolar moment of the system is described by $E_{\rm coupling} = -u_f m_{\rm d}(h_x h_y h_z + \alpha m)$, where u_f is a coefficient of coupling, and where we incorporate inter-domain coupling by a Weiss-like mean field term (α). $f_e \equiv h_x h_y h_z + \alpha m$ is the effective field.

We now consider the application of a magnetic field on the system that encourages the expansion of the $+m_\mathrm{d}$ domain i.e. it is energetically favourable to have both the domains align as $+m_\mathrm{d}$. In the absence of the pinning site, this domain wall slides over easily thus enabling the expansion of the domain. However, the pinning site obstructs this simple motion. In the spirit of Jiles and Atherton, we consider the energy required to overcome the pinning site to be equal (up to a proportionality constant, c_0) to the energy required to align the octupolar moment of $-m_\mathrm{d}$ with $+m_\mathrm{d}$ i.e.

$$\begin{aligned} E_\mathrm{cost} &= c_0\left(u_f m_\mathrm{d} f_e - (u_f m'_\mathrm{d} f_e)\right) \\ &= c_0\left(2u_f m_\mathrm{d} f_e\right) \ . \end{aligned} \tag{12}$$

This is the energy required to overcome a single pinning site. We now generalize the scenario where we have a collection of such pinning sites over a distance dx, and the magnetic field is applied such that the domain wall (of cross sectional area, A) is swept over that distance dx. If there exists an average density of pinning sites, n, over this volume $A\ dx$ and the average energy to overcome a site is $\langle E_\mathrm{cost}\rangle$ [where the averaging is performed over all the pinning sites] then the total energy dissipated through moving this domain wall through this mire of pinning sites is

$$E_\mathrm{total}(x) = \int_0^x \langle E_{cost}\rangle nAdx \ , \tag{13}$$

where $nAdx$ is the total number of pinning sites in the volume of interest. Since the change in the bulk octupolar moment (as the magnetic field moves the domain wall past the pinning site) is $dm_\mathrm{ir} = (m_\mathrm{d} - (-m_\mathrm{d}))\,Adx = 2m_\mathrm{d}Adx$, then we can replace the integrand in the above equation by

$$\begin{aligned} E_\mathrm{total}(x) &= \int_0^{m_{ir}} \frac{n\langle E_\mathrm{cost}\rangle}{2m_\mathrm{d}} dm_\mathrm{ir} \\ &= k\int_0^{m_{ir}} dm_\mathrm{ir} \ , \end{aligned} \tag{14}$$

where $k \equiv \frac{n\langle E_\mathrm{cost}\rangle}{2m_\mathrm{d}}$, which we take to be a constant. Although the derivation is quite involved, the final result physical makes sense in that the total work done in moving the domain wall past the pinning sites is proportional to the change in the bulk octupolar moment (associated with moving the domain wall).

Next, we consider the energy required to set up a bulk octupolar configuration, m_ir (under the presence of an effective field, f_e) which is the energy required to setup the ideal bulk octupolar moment in the absence of pinning sites, m, in an effective field, f_e, plus the energy required to overcome the domain wall,

$$\begin{aligned} -\int m_\mathrm{ir} df_e &= -\int m df_e + k\int dm_\mathrm{ir} \\ &= -\int m df_e + k\int \frac{dm_\mathrm{ir}}{df_e} df_e \end{aligned} \tag{15}$$

Collecting the integrands,

$$\int \left(m_\mathrm{ir} - m + k\frac{dm_\mathrm{ir}}{d\tilde{f}_e}\right) d\tilde{f}_e = 0 \ , \tag{16}$$

where we introduce the dummy variable of integration $\tilde{f}_e$. For magnetic fields being swept up from 0 to f_e, the bounds on the integrand are: $\int_\mathrm{up} \equiv \int_0^{f_e}$, while for sweeping down from 0 to $-f_e$ is from $\int_\mathrm{down} \equiv \int_0^{-f_e}$. Taking the derivative of the above Eq. 16 for the up sweep we get (by applying the Fundamental theorem of Calculus),

$$\begin{aligned} &\frac{d}{df_e}\left(\int_0^{f_e}\left(m_\mathrm{ir} - m + k\frac{dm_\mathrm{ir}}{d\tilde{f}_e}\right) d\tilde{f}_e = 0\right) \\ &\implies m_\mathrm{ir} = m - k\frac{dm_\mathrm{ir}}{df_e} \ . \end{aligned} \tag{17}$$

Similarly for the down sweeping magnetic field, we get (by changing variables in the integrand $\tilde{f}_e \to -\tilde{f}_e$),

$$\begin{aligned} &\frac{d}{df_e}\left(\int_0^{-f_e}\left(m_\mathrm{ir} - m + k\frac{dm_\mathrm{ir}}{d\tilde{f}_e}\right) d\tilde{f}_e = 0\right) \\ &\implies m_\mathrm{ir} = m + k\frac{dm_\mathrm{ir}}{df_e} \ . \end{aligned} \tag{18}$$

Thus, we obtain $m_\mathrm{ir} = m - \pm k\frac{dm_\mathrm{ir}}{df_e}$, where $\pm$ is for increasing and decreasing magnetic fields, respectively. Rearranging, we arrive at the following differential equation

$$\frac{dm_\mathrm{ir}}{df} = \frac{m - m_\mathrm{ir}}{\pm k - \alpha(m - m_\mathrm{ir})} \ . \tag{19}$$

Finally, we use $\frac{dm_\mathrm{ir}}{df} = \frac{dm_\mathrm{ir}}{dh}\frac{dh}{df}$ to obtain the so-called 'lag' equation i.e. the 'lag' $(m - m_\mathrm{ir})$ from the ideal value

$$\frac{dm_\mathrm{ir}}{dh} = \frac{m - m_\mathrm{ir}}{\pm k - \alpha(m - m_\mathrm{ir})}(3h^2) \ , \tag{20}$$

where k characterizes the pinning strength (encoding the number of pinning sites and the energy cost of overcoming a single pinning site), α is a constant describing the coupling between octupolar domains, and the sign $\pm$ applies respectively for increasing and decreasing magnetic fields. The experimentally relevant octupole moment is then given by

$$m_\mathrm{exp} = m_\mathrm{ir} + c(m - m_\mathrm{ir}) \ . \tag{21}$$

where c is a constant. The algorithm to determine the total macroscopic octupolar moment is straightforward. First we minimize the Landau free energy to obtain the ideal octupolar moment m. Next, we solve Eq. 20 for m_ir. Finally, we obtain m_exp by using Eq. 21.

SUPPLEMENTARY NOTE 8: ORIGIN OF CONDUCTION ELECTRON COUPLING TERM, γ_c

The quadratic γ_c term is constructed from purely symmetry arguments. This term is believed to arise from the independent coupling of the magnetic field and the lattice strain to the conduction electrons (and after integrating out the conduction electrons). The exact derivation of this term depends on the choice of the orbitals of the conduction electrons, but we can nonetheless provide a preliminary sketch of how this term arises.

For the sake of discussion, let us choose conduction electrons residing in p_x, p_y, p_z orbitals located on the diamond lattice sites. This construction has been employed in a recent work by some of the authors[3], where p_x, p_y, p_z are in fact molecular orbitals formed by the atoms in the Frank Kasper cage centred about each diamond lattice point. Constrained by the local T_d symmetry, we can write down the following coupling of the electrons to the magnetic field,

$$H_{\mathrm{mag},c} = a_1 \sum_{i,\{\alpha,\beta=\uparrow,\downarrow\}} \left(h_x\sigma^x_{\alpha\beta} c^\dagger_{i,x,\alpha} c_{i,x,\beta} + h_y\sigma^y_{\alpha\beta} c^\dagger_{i,y,\alpha} c_{i,y,\beta} + h_z\sigma^z_{\alpha\beta} c^\dagger_{i,z,\alpha} c_{i,z,\beta} \right)$$
$$+ a_2 \sum_{i,\{\alpha,\beta=\uparrow,\downarrow\}} \left(h_x\sigma^x_{\alpha\beta} \left[c^\dagger_{i,y,\alpha} c_{i,y,\beta} + c^\dagger_{i,z,\alpha} c_{i,z,\beta} \right] + h_y\sigma^y_{\alpha\beta} \left[c^\dagger_{i,x,\alpha} c_{i,x,\beta} + c^\dagger_{i,z,\alpha} c_{i,z,\beta} \right] + h_z\sigma^z_{\alpha\beta} \left[c^\dagger_{i,x,\alpha} c_{i,x,\beta} + c^\dagger_{i,y,\alpha} c_{i,y,\beta} \right] \right) \quad (22)$$

where $a_{1,2}$ are coupling coefficients, $\sum_i$ sums over the locations of the conduction electron in the lattice, the subscripts (x, y, z) of the fermionic operators (c) denote the orbital degree of freedom (p_x, p_y, p_z), the components of the external magnetic field are denoted by $h_{x,y,z}$, and $\sigma^{x,y,z}$ are 2×2 Pauli matrices representing the spin degree of freedom of the conduction electrons. We can similarly write down the direct coupling of the conduction electrons to the elastic strain tensor, which (again constrained by local T_d symmetry) is of the form,

$$H_{\mathrm{lattice},c} = a_3 \sum_{i,\{\alpha,\beta=\uparrow,\downarrow\}} \left(\epsilon_{xy} \left[c^\dagger_{i,x,\alpha} c_{i,y,\beta} + c^\dagger_{i,y,\alpha} c_{i,x,\beta} \right] + \epsilon_{yz} \left[c^\dagger_{i,y,\alpha} c_{i,z,\beta} + c^\dagger_{i,z,\alpha} c_{i,y,\beta} \right] + \epsilon_{xz} \left[c^\dagger_{i,x,\alpha} c_{i,z,\beta} + c^\dagger_{i,z,\alpha} c_{i,x,\beta} \right] \right) \delta_{\alpha\beta} \quad (23)$$

where a_3 is a coupling coefficient and ϵ is the familiar strain tensor. Together with a Slater-Koster tight-binding model of conduction electrons hopping on the diamond lattice sites $(H_{t,c})$, we have the following conduction electron Hamiltonian,

$$H_c = H_{t,c} + H_{\mathrm{mag},c} + H_{\mathrm{lattice},c} + H_{\mathrm{Kondo},c} \quad (24)$$

where $H_{\mathrm{Kondo},c}$ is a Kondo coupling term between the conduction electrons and local multipolar moments (as derived in Ref. 3). We thus have the following total partition function of multipolar moments and conduction electrons,

$$Z = \int \mathcal{D}[\tilde{\phi}, \phi, m, \bar{c}, c] e^{-S_c} e^{-S_{\tilde{\phi},\phi,m}} \quad (25)$$

where $S_c = \int d\tau \left[\bar{c}(\partial_\tau + H_c)c \right]$ is the conduction electron action, and $S_{\tilde{\phi},\phi,m}$ contains all the free energy terms of the multipolar moments. By formally integrating out the conduction electrons, we thus expect to obtain the γ_c coupling between the elastic strain and magnetic field. Clearly we will get such coupling, but precise determination of the coefficient is an intriguing future direction to pursue.

[1] Y. Nakanishi, M. Taniguchi, M. Nakamura, J. Hasegawa, R. Ohyama, M. Nakamura, M. Yoshizawa, M. Tsujimoto, and S. Nakatsuji. Elastic anomalies associated with two successive transitions of PrV_2Al_{20} probed by ultrasound measurements, Physica B: Condensed Matter **536**, 125 – 127 (2018).

[2] D. Jiles and D. Atherton. Theory of ferromagnetic hysteresis, Journal of Magnetism and Magnetic Materials **61**, 48 –

60 (1986).
[3] A. S. Patri, I. Khait, and Y. B. Kim. Emergent non-Fermi liquid phenomena in itinerant electron systems with multipolar local moments. Preprint at https://arxiv.org/abs/1904.02717 (2019).
